# The role of SHP/REV-ERBα/CYP4A axis in the pathogenesis of alcohol-associated liver disease

**DOI:** 10.1172/jci.insight.140687

**Published:** 2021-08-23

**Authors:** Zhihong Yang, Rana V. Smalling, Yi Huang, Yanchao Jiang, Praveen Kusumanchi, Will Bogaert, Li Wang, Don A. Delker, Nicholas J. Skill, Sen Han, Ting Zhang, Jing Ma, Nazmul Huda, Suthat Liangpunsakul

**Affiliations:** 1 Division of Gastroenterology and Hepatology, Department of Medicine, Indiana University School of Medicine, Indianapolis, Indiana, USA.; 2 Vanderbilt University Medical Center, Nashville, Tennessee, USA.; 3 Department of Physiology and Neurobiology, University of Connecticut, Storrs, Connecticut, USA.; 4 Department of Internal Medicine, Section of Digestive Diseases, Yale University, New Haven, Connecticut, USA.; 5 Divisions of Gastroenterology, University of Utah, Salt Lake City, Utah, USA.; 6 Department of Surgery, Indiana University School of Medicine, Indianapolis, Indiana, USA.; 7 Roudebush Veterans Administration Medical Center, Indianapolis, Indiana, USA.; 8 Department of Biochemistry and Molecular Biology, Indiana University School of Medicine, Indianapolis, Indiana, USA.

**Keywords:** Hepatology, Fatty acid oxidation, Mouse models, Transcription

## Abstract

Alcohol-associated liver disease (ALD) represents a spectrum of histopathological changes, including alcoholic steatosis, steatohepatitis, and cirrhosis. One of the early responses to excessive alcohol consumption is lipid accumulation in the hepatocytes. Lipid **ω**-hydroxylation of medium- and long-chain fatty acid metabolized by the cytochrome P450 4A (CYP4A) family is an alternative pathway for fatty acid metabolism. The molecular mechanisms of CYP4A in ALD pathogenesis have not been elucidated. In this study, WT and *Shp^−/−^* mice were fed with a modified ethanol-binge, National Institute on Alcohol Abuse and Alcoholism model (10 days of ethanol feeding plus single binge). Liver tissues were collected every 6 hours for 24 hours and analyzed using RNA-Seq. The effects of REV-ERB**α** agonist (SR9009, 100 mg/kg/d) or CYP4A antagonist (HET0016, 5 mg/kg/d) in ethanol-fed mice were also evaluated. We found that hepatic *Cyp4a10* and *Cyp4a14* expression were significantly upregulated in WT mice, but not in *Shp^−/−^* mice, fed with ethanol. ChIP quantitative PCR and promoter assay revealed that REV-ERB**α** is the transcriptional repressor of *Cyp4a10* and *Cyp4a14*. *Rev-Erb***α***−/−* hepatocytes had a marked induction of both *Cyp4a* genes and lipid accumulation. REV-ERB**α** agonist SR9009 or CYP4A antagonist HET0016 attenuated *Cyp4a* induction by ethanol and prevented alcohol-induced steatosis. Here, we have identified a role for the SHP/REV-ERB**α**/CYP4A axis in the pathogenesis of ALD. Our data also suggest REV-ERB**α** or CYP4A as the potential therapeutic targets for ALD.

## Introduction

Excessive alcohol consumption is the leading cause of several adverse health outcomes, including alcohol-associated liver disease (ALD; ref. 1–3). ALD comprises a spectrum of histopathological changes and clinical disorders in patients with acute and chronic alcohol consumption, ranging from alcoholic steatosis, steatohepatitis, advanced fibrosis, and cirrhosis (4, 5).

The pathogenesis of alcohol-induced liver injury is complex involving the alterations of lipid metabolism, oxidative stress, inflammatory signaling pathway, and disruption of circadian clock machinery (6–8). Several genes regulating lipid metabolism are under the control of the cell-autonomous circadian clock (9–11). The molecular clock, consisting of a series of autoregulatory transcriptional feedback loops, generates a rhythmicity controlling metabolic pathway over the course of the day by a self-sustainable pacemaker through the input from environmental cues (9, 11). The genes of brain and muscle ARNT-like 1 (*BMAL1*) and circadian locomotor output cycles kaput (*CLOCK*) encode basic helix-loop-helix; per-arnt-single-minded (bHLH-PAS) proteins, BMAL1, and CLOCK, which are part of the positive feedback loop. The CLOCK:BMAL1 heterodimer instigates the transcription by binding to specific DNA elements in the promoters of the target genes, such as cryptochrome (*CRY*) and period (*PER*), forming the negative limb of the feedback loop. The resulting CRY and PER proteins subsequently inhibit CLOCK:BMAL1 transcriptional activity. The CLOCK:BMAL1 dimers also initiate the transcription of an interconnecting loop, which involves the E-box mediated transcription of the orphan nuclear-receptor genes retinoid orphan nuclear receptor α/β (RORα/β) and REV-ERBα/β. The ROR and REV-ERB proteins can compete for retinoic acid-related orphan receptor response element binding sites within the *BMAL1* promoter in which the ROR and REV-ERB proteins initiate and inhibit *BMAL1* transcription, respectively. Disturbance in the circadian machinery pathway such as dysregulation of REV-ERB can lead to an elevation of serum triglyceride and hepatic steatosis (12–14).

The small heterodimer partner (SHP, NR0B2) functions as a transcriptional repressor and is critical in regulating hepatic metabolism (15). Recent studies indicate that multiple genes in the circadian pathways such as *CLOCK* and *REV-ERB*α are under the regulation of SHP (16, 17). We previously reported the effect of ethanol feeding on intrahepatic clock machinery and the critical role of SHP and REV-ERBα in controlling rhythmic expression of CCAAT-enhancer-binding protein homologous protein, a transcription factor in ER stress response in ethanol-fed mice (18).

The cytochrome P450 (CYP) superfamily is a group of heme-containing proteins with multiple functions, including the metabolism of xenobiotics such as alcohol, drugs, toxins, carcinogens, and endogenous substrates, like fatty acids and steroids (19). CYP-dependent ω-hydroxylation is the third oxidation reaction that transforms the terminal methyl group of a hydrophobic aliphatic chain into a more polar alcohol metabolite (20). The CYP4 family consists of 11 subfamilies (CYP4A-CYP4M), which encode constitutive and inducible isozymes (21). Murine *Cyp4a10* and *Cyp4a14* (homologous to *CYP4A22* and *CYP4A11* in human, respectively) are highly expressed in the liver (22) and are known to convert the arachidonic acid to its metabolite 20-hydroxyeicosatetraenoic acid (20-HETE), which regulates inflammatory processes through the generation of ROS (23). Inhibition of *Cyp4a14* was recently reported to attenuate hepatic steatosis and fibrosis (20, 24). However, the role of CYP4A in the pathogenesis of alcohol-induced liver injury is largely unknown.

In this study, we performed RNA-Seq, comparing hepatic gene expression in WT and *Shp^−/−^* mice fed with chronic alcohol plus binge model. We found that the stimulatory effect of ethanol on both *Cyp4a10* and *Cyp4a14* was significantly reduced in *Shp^−/−^* mice. Using the luciferase and ChIP assay, we identified a potentially novel SHP/REV-ERBα/CYP4A axis in the pathogenesis of alcohol-induced liver injury. Furthermore, we found that pharmacological intervention targeting REV-ERBα and CYP4A attenuated alcohol-induced liver injury.

## Results

### Hepatic Cyp4a10 and Cyp4a14 were substantially attenuated in Shp^−/−^ mice fed with the ethanol plus binge model.

We have previously shown that excessive alcohol use disrupts hepatic circadian clock machinery leading to alterations in intrahepatic lipid metabolism and hepatic steatosis (11). However, the mechanism underlying this observation has not yet been elucidated. Based on our previous studies that Shp regulates circadian clock regulator*,* we thus reasoned that Shp may be a key factor regulating the hepatic circadian clock and the effect of alcohol on hepatic phenotypes (16, 25). Therefore, we fed WT and *Shp^−/−^* mice with control or an ethanol-containing diet using the ethanol plus binge model, as previously described (26). Liver tissues were collected at the end of the experiments approximately 9 hours (Zeitgeber time 12 [ZT 12]) after oral gavage (with either maltose or ethanol), and then at every 6 hours (ZT 18, ZT 0, and ZT 6) over a 24-hour period (*n =* 3/treatment group/ZT time point). As we previously reported, hepatic steatosis was remarkably increased in ethanol-fed WT mice but ameliorated in ethanol-fed *Shp^−/−^* mice (18).

**Figure 1:**
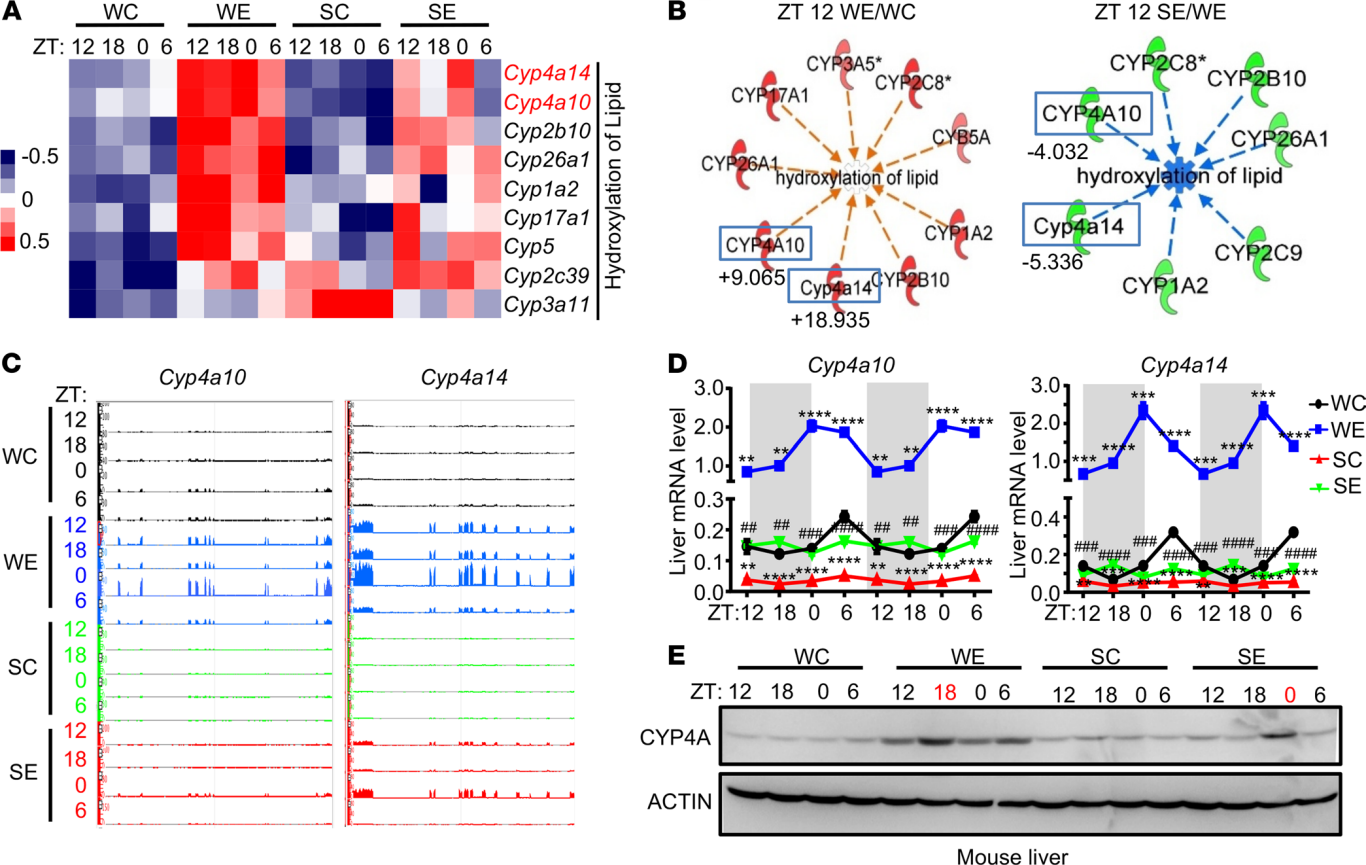
Activation of hepatic Cyp4a10 and Cyp4a14 in mice fed with ethanol plus binge model was attenuated in Shp−/− mice. (**A**) Heatmap of RNA-Seq analysis from WT and *Shp^−/−^* mice treated with or without ethanol plus binge model (*n =* 3/group/ZT time point). SC, *Shp^−/−^* control; SE, *Shp^−/−^* treated with ethanol; WC, WT control; WE, WT treated with ethanol; ZT, Zeitgeber time. (**B**) IPA-generated hydroxylation of lipid network using the data from the liver tissue at ZT 12 from each experimental group using Ingenuity Pathway Analysis (IPA). Green, down-regulated in indicated comparisons; numbers under the blue box, fold changes; red, upregulated in indicated comparisons. (**C**) Genome browser view of RNA-Seq reads in the *Cyp4a10* and *Cy4a14* loci. (**D**) qPCR validation of *Cyp4a10* and *Cyp4a14* mRNAs expression. *P < 0.05, **P < 0.01, ***P < 0.001, ****P < 0.0001 versus WC; ^#^*P <* 0.05, ^##^*P <* 0.01, ^###^*P <* 0.001, ^####^*P <* 0.0001 versus WE. Two-way ANOVA. (**E**) Western blot analysis of CYP4A expression. The ZT time point highlight in red is when the CYP4A expression reached the peak in that group (*n =* 3/group/ZT time point).

To determine the mechanisms related to the protective effects of SHP on alcohol-induced liver injury, we performed RNA-Seq from liver tissues, which were collected at each ZT time point from mice in each group (Figure 1A). Log-transformed fragments per kilobase of transcript per million mapped reads (FPKM) were used to generate heatmaps in Cluster (version 3.0) and Java Tree View (version 3.0). The heatmap representing the upregulated genes in ethanol-fed WT mice (WE) compared with WT controls (WC) showed a significant increase in the Cyp family expression, including *Cyp4* and *Cyp2* genes**, **which was markedly decreased in Shp^−/−^ mice fed with ethanol (SE), notably at ZT 6 (Figure 1A). ZT 12, approximately 9 hours after alcohol oral gavage, was the time when we observed the level of hepatic transaminases at their peak (26). Therefore, we selected the representative data from ZT 12 for subsequent bioinformatic analysis. Ingenuity Pathway Analysis (IPA) using the RNA-Seq data from ZT 12 showed a consistent association between several CYP genes, including *Cyp4a10* and *Cyp4a14,* which were activated in WE (compared with WC) and inhibited in SE (compared with WE; Figure 1B). Those CYP genes belonged to the lipid hydroxylation pathway, and the specific activation *z* scores demonstrated an increasing trend of lipid hydroxylation pathway in WE compared with WC (at all ZT time points) and a reduction in SE compared with WE (a *z* score of more than ± 2 is considered significant) (Supplemental Figure 1; supplemental material available online with this article; https://doi.org/10.1172/jci.insight.140687DS1). Our data indicated that the activation of the lipid hydroxylation pathway by ethanol was markedly reduced in *Shp* deficiency.

To further validate the increase in the expression of CYP4A in our ethanol model, we determined the RNA-Seq peak of the expression for *Cyp4a10* and *Cyp4a14* at each ZT time point in 4 experimental groups (Figure 1C). We found an increase in the peak intensity of *Cyp4a10* and *Cyp4a14* in WE (shown in blue) when compared with WC, and their expression was markedly decreased in the SE group (shown in red). We next determined the mRNA as well as the protein expression using qPCR and Western blot analysis, respectively (Figure 1, D and E). We found an increase in *Cyp4a10* and *Cyp4a14* mRNA expression and total CYP4A protein levels in WE compared with WC. The expression also exhibited rhythmicity across the ZT time point (Figure 1, D and E). Interestingly, the circadian pattern and the mRNA expression of both *Cyp4a10* and *Cyp4a14* were blunted and the peaks were shifted in the SE group (Figure 1, D and E).

We also explored the rhythmicity of core clock genes and found the expression of *Per2, Bmal,* and *Clock* in the opposite direction compared with that of *Rev-Erb*α across the 24-hour period (Supplemental Figure 2A). Interestingly, the deficiency of Shp did not significantly alter the rhythmicity of Rev-Erbα, comparing its hepatic expression in WE versus SE (Supplemental Figure 2A). We also determined the hepatic rhythmicity of other CYP4A subfamilies (*Cyp4a12a* and *Cyp4a12b*; Supplemental Figure 2B) and observed a differential effect of alcohol feeding on *Cyp4a10* and *Cyp4a14* compared with that of *Cyp4a12a* and *Cyp4a12b*. Overall, CYP4A protein expression was significantly increased after alcohol feeding protein (Figure 1E), in the same direction with an increase in *Cyp4a10* and *Cyp4a14* transcripts. Taken together, our data suggest that ethanol regulates CYP4A expression through SHP.

### REV-ERBα is the potential circadian transcriptional regulator of both Cyp4a10 and Cyp4a14.

**Figure 2:**
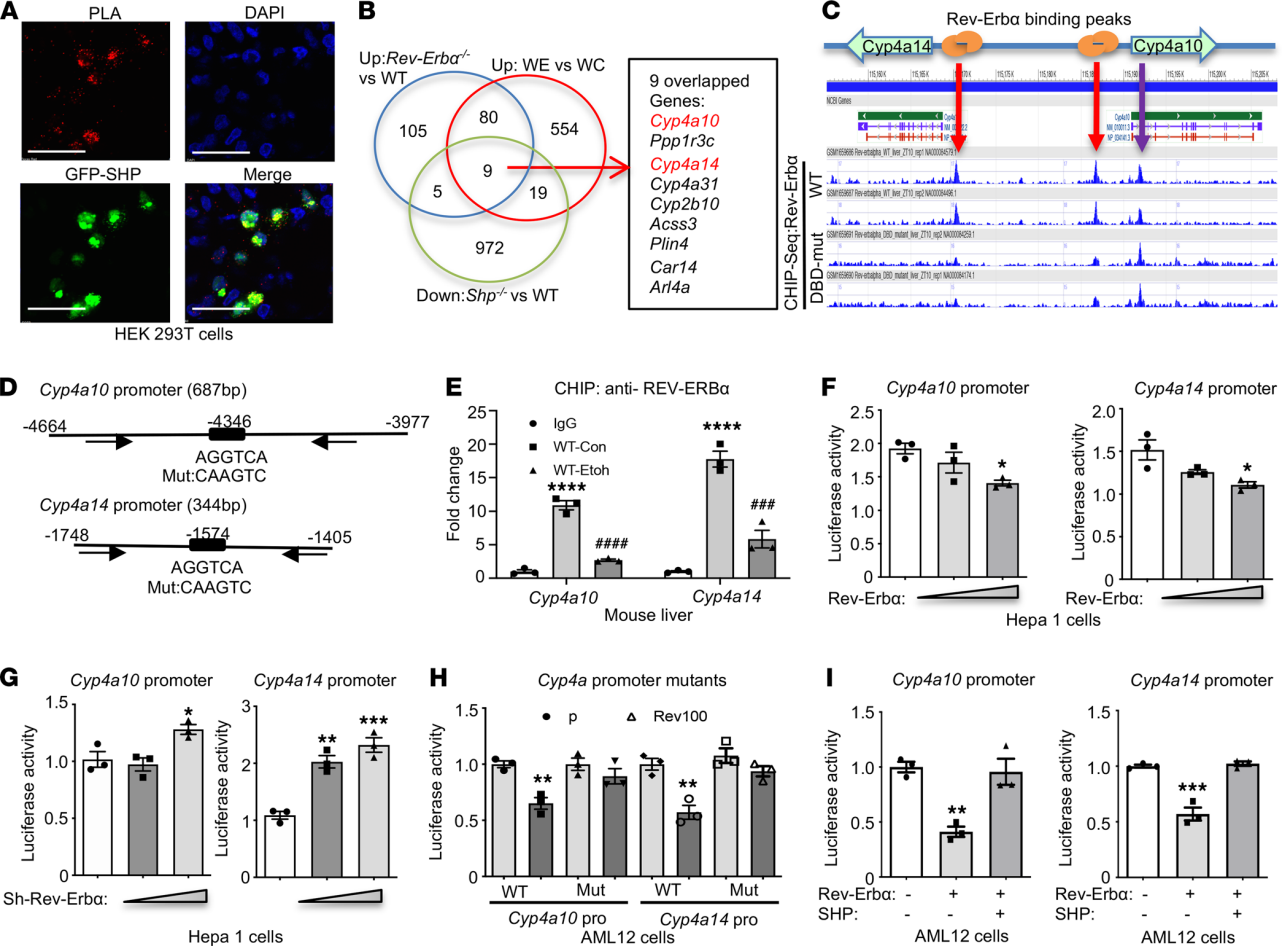
REV-ERBα is the potential transcriptional regulator of Cyp4a10 and Cyp4a14. (**A**) Proximity ligation assay (PLA, red) demonstrated the interaction between GFP-SHP (green) and FLAG-REV-ERBα proteins in HEK293T cells. Scale bar: 100 μm. (**B**) Three RNA-Seq data sets, *Rev-Erb*α*−/−* versus WT (GSE59486, GSE59460), *Shp^−/−^* versus WT (GSE43893), and WE versus WC (ZT 12), were integrated to identify overlapping genes, which were coregulated by each pathway. Venn diagram indicated 9 overlapping genes, including both *Cyp4a10* and *Cyp4a14* (red). WC, WT control; WE, WT treated with ethanol. (**C**) ChIP-Seq (GSE67962) revealed the location of REV-ERBα binding peaks on *Cyp4a10* and *Cyp4a14* promoters in mouse livers (red arrows). The mutated REV-ERBα DNA-binding domain (DBD-mut) served as negative controls. The binding peak indicated by the purple arrow could be the binding independent from REV-ERBα DNA-binding domain. (**D**) The diagram of *Cyp4a10* and *Cyp4a14* promoter with distance from transcription start site (TSS) and REV-ERBα binding sites. Black arrows indicated location of the ChIP primers. Mut, mutant on the REV-ERBα binding site in the promoter constructs. (**E**) ChIP assay with anti-REV-ERBα Ab or IgG (negative control) from mouse liver at ZT 12. WT-Con, WT control; WT-Etoh, WT-ethanol. ****P < 0.0001 versus IgG; ^###^*P <* 0.001, ^####^*P <* 0.0001 versus WT-Con. One-way ANOVA. (**F**) Luciferase assay with *Cyp4a10* or *Cyp4a14* promoter and cotransfected with 0, 50, or 100 ng/well of REV-ERBα plasmids in 24-well plates. *P < 0.05, **P < 0.01 versus control. One-way ANOVA. (**G**) Luciferase assay with indicated promoters and cotransfected with 0, 50, 100 ng/well of pLKO-shRNA-REV-ERBα plasmids (sh-Rev-Erbα). *P < 0.05, **P < 0.01, ***P < 0.001 versus control. One-way ANOVA. (**H**) Luciferase assay with indicated promoters and cotransfected with 100 ng/well pcDNA3 (p) or REV-ERBα (Rev100) plasmids. **P < 0.01 versus pcDNA3. Two-tailed Student’s *t* test. (**I**) Luciferase assay with indicated promoters and plasmids (100 ng/each). **P < 0.01, ***P < 0.001 versus control. One-way ANOVA.

The circadian rhythmicity of *Cyp4a10* and *Cyp4a14* in ethanol-fed mice (Figure 1, D and E) led us to hypothesize that their expression is under the control of the circadian transcriptional regulator. We previously reported the interaction between SHP and REV-ERBα in the pathogenesis of alcohol-induced liver injury (18). This raised an interesting question as to whether SHP/REV-ERBα acts as a transcription regulator of CYP4A. To visualize the interaction, we first performed the Duolink proximity ligation assay (PLA) to determine the protein interactions between SHP and REV-ERBα. FLAG-tagged REV-ERBα and GFP-tagged SHP were coexpressed in HEK293T cells for 24 hours, then the antibodies for anti-mouse FLAG and anti-rabbit GFP were used following the manufacturer’s protocol (PLA kit, MilliporeSigma). We found the interaction between SHP and REV-ERBα, located primarily in the nucleus (Figure 2A and Supplemental Figure 3A). To determine if *Cyp4a10* and *Cyp4a14* are the targets of REV-ERBα, we performed bioinformatic analyses using the online Gene Expression Omnibus database. The upregulated genes were gated with a fold change of greater than 1.3 in both GRO-Seq (GSE59486) and microarray (GSE59460, FDR <0.1) of *Rev-Erbα−/−* mice experiments (27). We then overlapped these genes with RNA-Seq from *Shp^−/−^* mice liver (GSE43893) and our RNA-Seq data in WE (using the data from ZT 12; Figure 2B). Venn diagram showed that only 9 genes were overlapped using the data from these 3 databases. Among them, we found upregulation of both *Cyp4a10* and *Cyp4a14* (Figure 2B, shown in red). GW4064 is the agonist for the farnesoid X receptor, a transcriptional activator of SHP. We did not observe the alteration between SHP and REV-ERBα interaction with GW4064 in the presence or absence of ethanol (Supplemental Figure 3B).

To determine if *Cyp4a10* and *Cyp4a14* are transcriptionally regulated by REV-ERBα, we analyzed the ChIP-Seq data (GSE67962) and found the possible binding of REV-ERBα on *Cyp4a10* and *Cyp4a14* promoters in mouse livers (Figure 2C). These bindings were markedly diminished when the REV-ERBα DNA-binding domain was mutated (DBD-Mut, Figure 2, C and D, and Supplemental Figure 4). To confirm our bioinformatic analysis, we next performed a ChIP assay using the anti-REV-ERBα antibody in liver samples from the WC and WE group (at ZT 12). The primer design is provided in Figure 2D and Supplemental Figure 5. We found that REV-ERBα positively binds to both *Cyp4a10* and *Cyp4a14* promoters, and the bindings were significantly inhibited in ethanol-fed mice (Figure 2E). Taken together, our data suggested that REV-ERBα transcriptionally regulated *Cyp4a10* and *Cyp4a14* through direct binding at their promoter regions. We next performed the luciferase assay to confirm the functional regulation of *Cyp4a10* and *Cyp4a14* promoters by REV-ERBα. We cloned both promoters base on the predicted REV-ERBα binding region (Figure 2D and Supplemental Figure 5) and transfected them into Hepa1 cells. We found the inhibition of both *Cyp4a10* and *Cyp4a14* promoter activities when we cotransfected the cells with REV-ERBα plasmid (Figure 2F); whereas knocking down REV-ERBα using shRNA significantly increased the promoter activities (Figure 2G). When we mutated the potential REV-ERBα binding (Figure 2D), the inhibitory effect of REV-ERBα on *Cyp4a10* and *Cyp4a14* promoter activities was ameliorated (Figure 2H). To determine the role of SHP in the regulation of CYP4A by REV-ERBα, we overexpressed SHP in the presence of REV-ERBα and found that SHP attenuated the inhibitory effect of both promoters by REV-ERBα (Figure 2I). Consistent with our promoter assay in vitro, we found a reduction in mRNA expression of *Cyp4a10* and *Cyp4a14* when we overexpressed REV-ERBα plasmid in vivo (Supplemental Figure 6A). On the contrary, the mRNA and protein expression of CYP4As were increased when we knocked down hepatic REV-ERBα using AAV8-U6-shREV-ERBα (Supplemental Figure 6, B and C). Last, we also observed the rhythmicity of hepatic *Cyp4a10* and *Cyp4a14* mRNA expression in the opposite direction compared with that in *Rev-Erb*α across the 24-hour period (Figure 1D and Supplemental Figure 2A). Taken together, our data demonstrated REV-ERBα as the transcriptional repressor of *Cyp4a10* and *Cyp4a14*.

### REV-ERBα deficiency remarkably induced both Cyp4a10 and Cyp4a14 expression, promoting lipid accumulation and oxidative stress.

**Figure 3:**
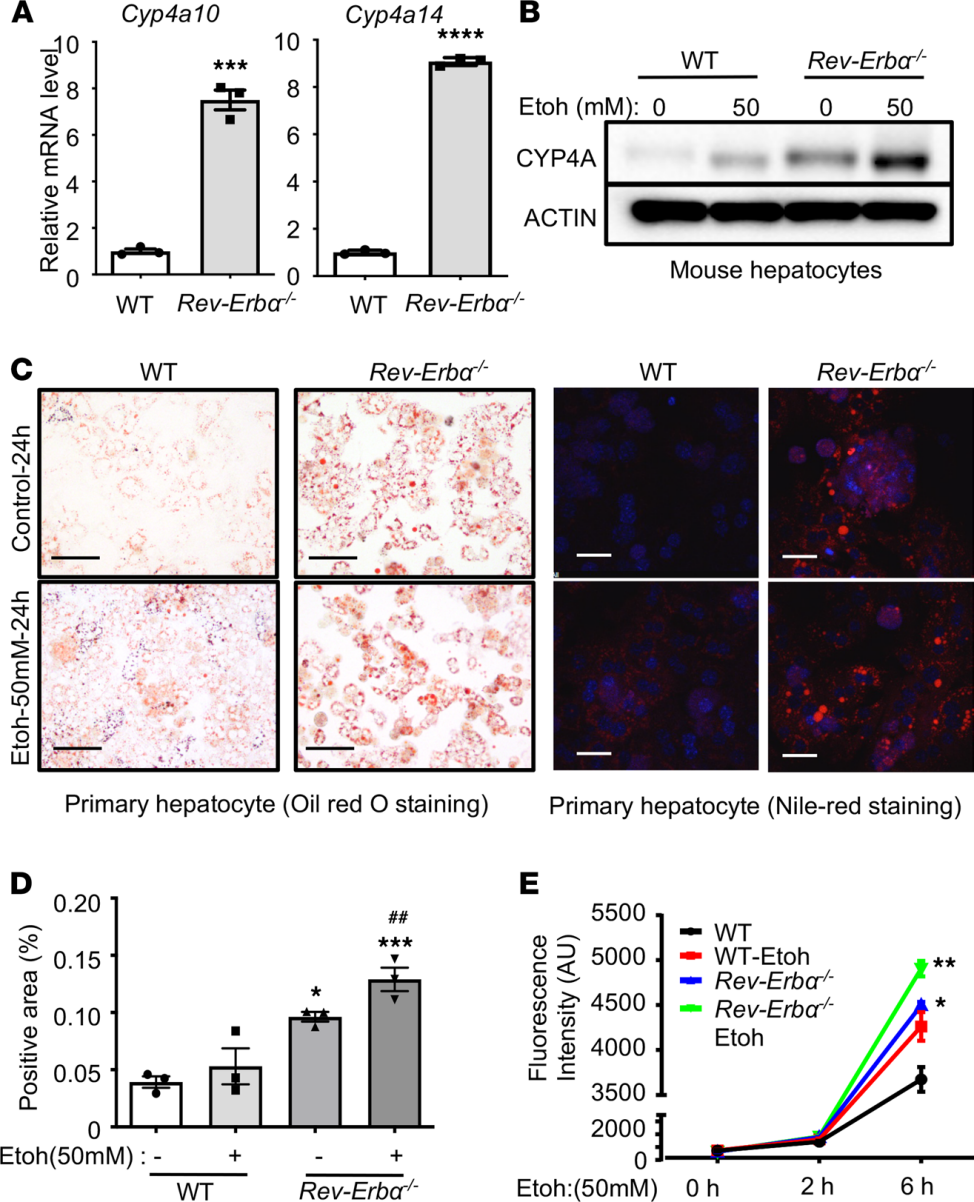
Deficiency of REV-ERBα largely induced both Cyp4a10 and Cyp4a14 expression, promoted lipid accumulation and oxidative stress. (**A**) qPCR analysis of hepatic *Cyp4a10* and *Cyp4a14* mRNAs in WT or *Rev-Erb*α*−/−* mice ***P < 0.001; ****P < 0.0001 versus WT. Two-tailed Student’s *t* test. (**B**) Western blot analysis of CYP4A protein from WT and *Rev-Erb*α*−/−* primary hepatocytes treated with or without 50 mM ethanol (Etoh) for 24 hours. (**C**) Oil Red O (left) and Nile Red (right) staining in primary hepatocytes of WT or *Rev-Erb*α*−/−* mice treated with or without 50 mM ethanol for 24 hours. Scale bar: 100 μm. (**D**) The quantification of the positive area (% to total area) from Nile Red staining (right). *P < 0.05, **P < 0.01 versus WT-E(-); ^##^*P <* 0.05 versus WT-E(+). One-way ANOVA. (**E**) ROS generation from WT and *Rev-Erb*α*−/−* primary hepatocytes treated with or without 50 mM Ethanol for indicated times. *P < 0.05, **P < 0.01 versus WT. One-way ANOVA.

To further confirm the regulation of CYP4As by REV-ERBα, we next determined the hepatic *Cyp4a10* and *Cyp4a14* expression in *Rev-Erb*α*−/−* mice. We found a significant increase in *Cyp4a10* (approximately 8-fold) and *Cyp4a14* (approximately 6-fold) in *Rev-Erb*α*−/−* mice (Figure 3A and Supplemental Figure 2D). Interestingly, we did not observe the change in the expression of other CYP4A subfamilies in WT and *Rev-Erb*α*−/−* mice, suggesting the specific regulation of REV-ERBα on *Cyp4a10* and *Cyp4a14* (Supplemental Figure 2D). We also utilized the public database (GSE79087) to determine the effect of REV-ERBα deletion on the hepatic rhythmicity of CYP4A. The results are illustrated in Supplemental Figure 2D.

We also found an increase in CYP4A protein expression when we treated primary hepatocytes with ethanol (50 mM for 24 hours), and its expression was significantly enhanced by *Rev-Erb*α** deficiency (Figure 3B). We observed an increase in lipid accumulation, especially in ethanol-treated *Rev-Erb*α*−/−* primary hepatocytes, which was examined by Oil Red O and Nile Red staining (Figure 3, C and D). Oxidative stress is implicated in alcohol-induced liver injury (28). We next measured the ROS generation in the hepatocytes from WT and *Rev-Erb*α*−/−* primary hepatocytes treated with and without ethanol (50 mM) at the indicated times. We found that ethanol treatment significantly augmented ROS generation in *Rev-Erb*α*−/−* hepatocytes (Figure 3E). Taken together, our data showed that ethanol significantly increased CYP4A expression, lipid accumulation, and oxidative stress in REV-ERBα–deficient hepatocytes.

### REV-ERBα and CYP4A as the therapeutic targets for alcohol-induced liver injury.

We next asked if intervention to activate REV-ERBα or inhibit CYP4A ameliorates alcohol-induced liver injury. SR9009 and SR9011 are synthetic REV-ERB agonists, which are able to activate both REV-ERBα and REV-ERBβ (29) and regulate lipid metabolism (30). Between these 2 agonists, SR9009 demonstrated higher potency on REV-ERBα than REV-ERBβ (29). N-Hydroxy-N′-(4-butyl-2-methylphenyl)-formamidine (HET0016) is the antagonist for CYP4A, which can selectively inhibit the biosynthesis of 20-HETE (ref. 31). We selected these compounds for the subsequent experiments.

**Figure 4:**
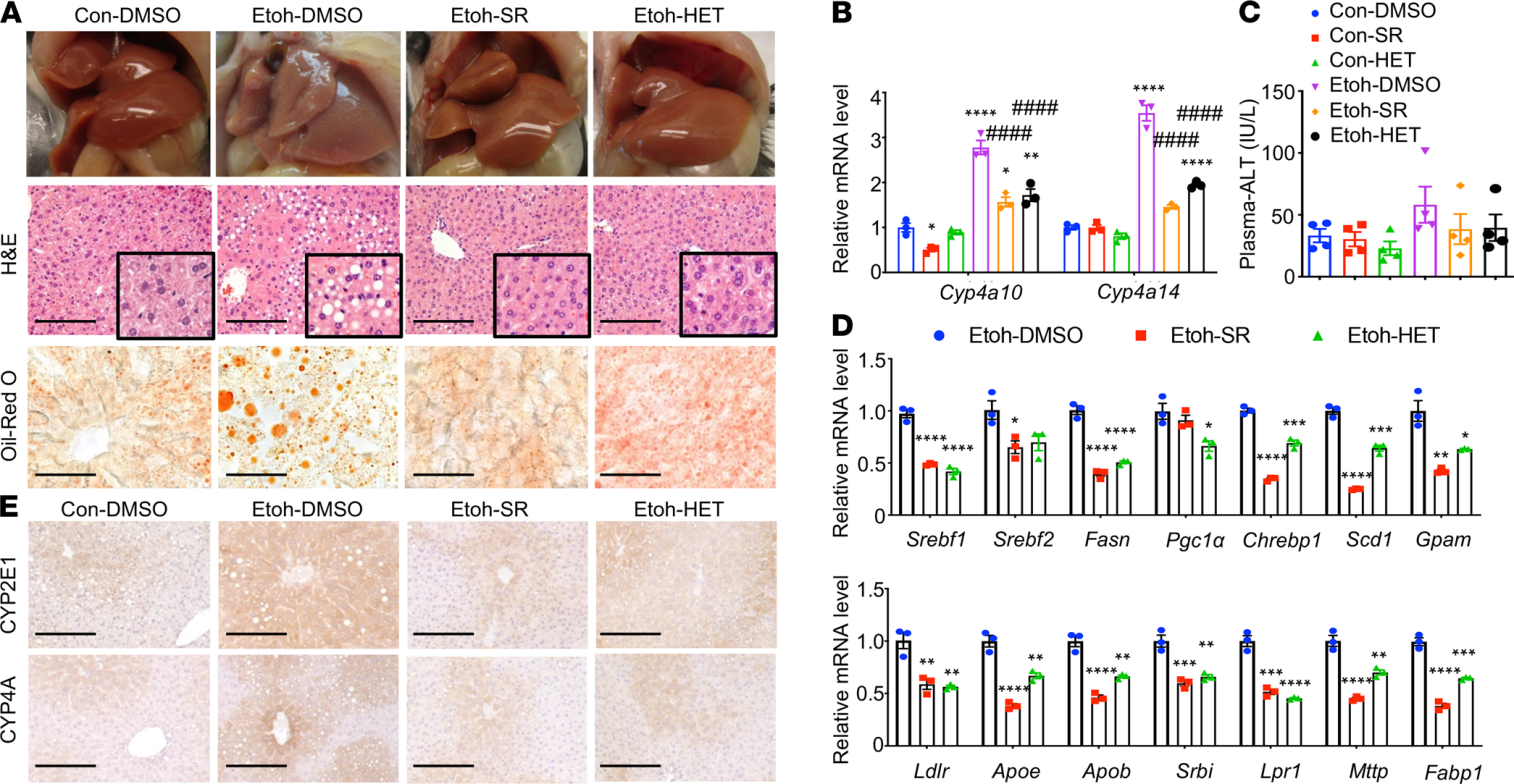
Treatment of REV-ERBα agonist SR9009 or CYP4A antagonist HET0016 substantially improved alcoholic steatosis and alcohol-induced liver injury. (**A**) Gross appearance (top), H&E staining (middle), and Oil Red O staining (bottom) of the livers of mice treated with SR9009 (100 mg/kg/d) or HET0016 (5 mg/kg/d) from days 1–11 over the course of ethanol feeding (*n =* 4/group). (**B**) QPCR analysis of *Cyp4a10* and *Cyp4a14* mRNAs. *P < 0.05, **P < 0.01, ****P < 0.0001 versus Con-DMSO; ^####^*P <* 0.001 versus Etoh-DMSO. One-way ANOVA. (**C**) Plasma ALT in indicated groups. Ns versus Con-DMSO or Etoh-DMSO. One-way ANOVA. (**D**) QPCR analysis of genes related to lipid metabolism (top panel) and lipid delivery (bottom panel). *P < 0.05, **P < 0.01, ***P < 0.001, ****P < 0.0001 versus Etoh-DMSO. One-way ANOVA. (**E**) IHC staining of CYP2E1 or CYP4A in each experimental group. Con, control; Etoh, ethanol; HET, CYP4A antigonist-HET0016; SR, REV-ERBα agonist-SR9009. Scale bar: 200 μm.

As described in Methods and Supplemental Figure 7, SR9009 and HET0016 were administrated during the alcohol feeding period. We found the protective effects of these 2 compounds against alcoholic steatosis in our mouse model (Figure 4). Morphologically, the livers from mice treated with Etoh plus vehicle (DMSO) group were pale; whereas those treated with Etoh plus SR9009 or HET0016 appeared to have a normal color, similar to those as observed in controls (Figure 4A, top, and Supplemental Figure 8A). Histological analysis with H&E and Oil Red O staining also confirmed the protective effects of these 2 compounds on alcohol-induced lipid accumulation (Figure 4A, middle and bottom).

The administration of SR9009 and HET0016 significantly decreased the mRNA and protein expression of both *Cyp4a10* and *Cyp4a14*, serum ALT, and genes involved in lipid metabolism compared with DMSO-treated mice in ethanol-fed groups (Figure 4, B–D, and Supplemental Figure 8, B and C). As we observed the increase in oxidative stress in *Rev-Erb*α–deficient hepatocytes, we also determined the expression of CYP2E1, an alcohol metabolizing enzyme, which is responsible for oxidative stress generation (32). We found that hepatic CYP2E1 protein expression was significantly reduced in ethanol-fed mice treated with either SR9009 or HET0016 (Figure 4E upper panel and Supplemental Figure 8D). Our results showed that REV-ERBα and CYP4A are the therapeutic targets for alcohol-induced liver injury.

### Activation of REV-ERBα with agonist SR9009 partially restored the effect of ethanol on hepatic metabolomic profiles.

**Figure 5:**
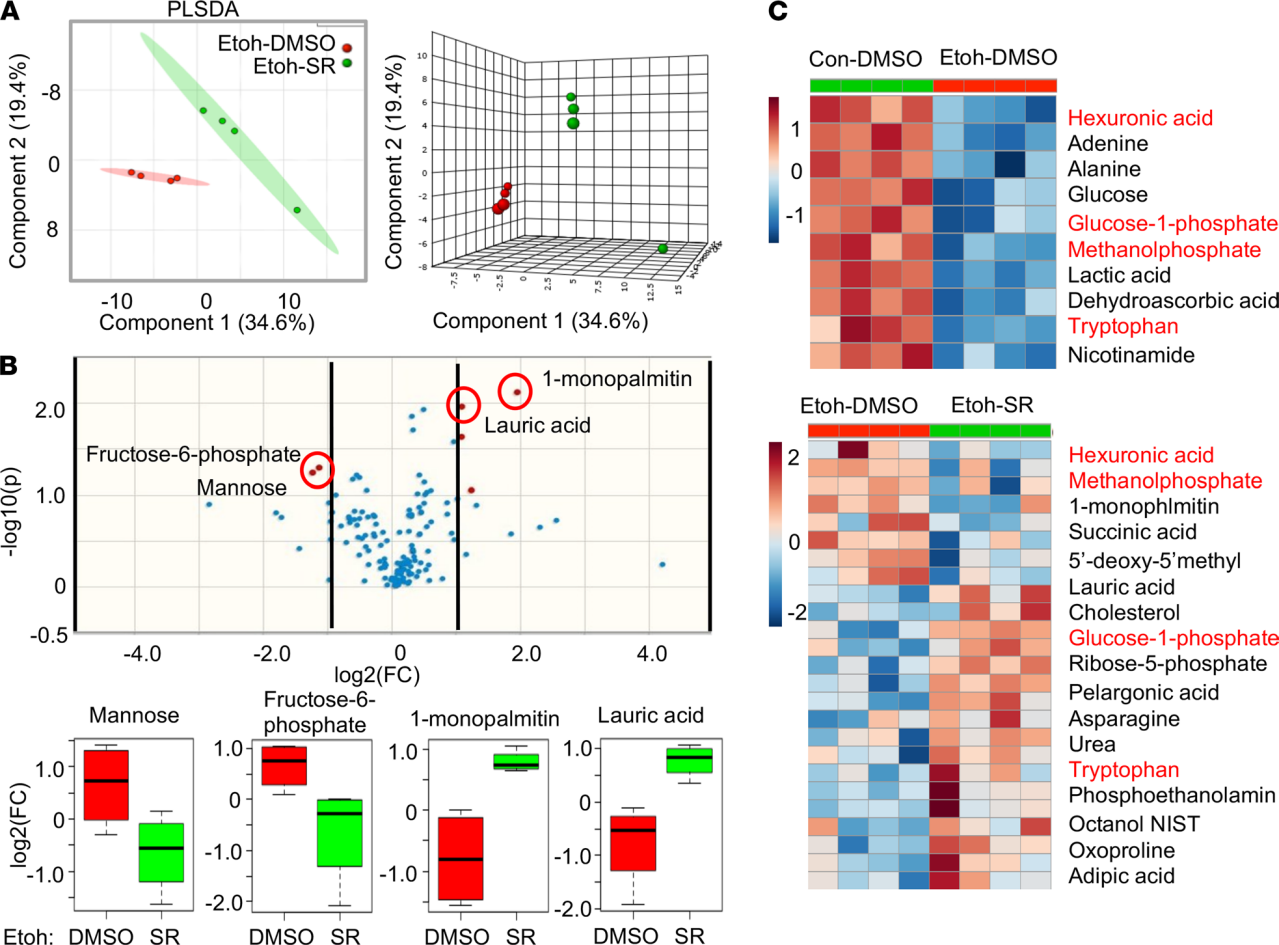
Treatment with REV-ERBα agonist SR9009 partially restored the dysregulation of hepatic metabolic profiles in ethanol-fed mice. (**A**) The predominance of partial least squares-discriminant analysis (PLS-DA) scores plots in 2D (left) and 3D (right) format. The ovals filled with different color indicated 95% CI Hotelling’s ellipses. (**B**) Volcano plot (upper) indicated the significantly changed metabolites. The red dots represent metabolites with a *P* value ≤0.05 and log2(FC) > or < 1. FC: fold changes. Red-circled dots were selected metabolites, shown in box plots (lower). (**C**) Heatmap showing differential abundance of metabolites in ethanol-fed mice (Etoh-DMSO) versus Control (Con-DMSO) (upper) and ethanol-fed mice treated with SR9009 (Etoh-SR) versus ethanol-fed mice (Etoh-DMSO). Red label indicated metabolites decreased in ethanol-fed mice, which were restored with SR9009 treatment.

Alterations in hepatic metabolites have been described in ethanol-fed mice (33). To expand our knowledge on the global alterations of the metabolites after the activation of REV-ERBα (using SR9009) with subsequent inhibition of CYP4A (which plays role in ω oxidation of fatty acid), we next carried out the experiments using untargeted metabolomic analysis. The 2- and 3-dimensional partial least squares discriminant plots based on principal component analysis of the metabolomic data were constructed (Figure 5A). We found a clear separation in hepatic metabolites in ethanol and ethanol with SR9009 groups, which indicated a significant difference in metabolic profiles between these 2 groups. Using the volcano plot (Figure 5B, top panel), we found that the metabolites with the most significant fold changes were 1-monopalmitin and lauric acid (upregulated) and fructose-6-phosphate and mannose (downregulated, Figure 5B). Interestingly, one-half of the top 10 downregulated metabolites in the Etoh group were restored upon treatment with SR9009 (Figure 5C). The metabolite set enrichment analysis revealed the significant change in metabolites between Etoh and Etoh plus SR9009 groups, mostly belong to the glucose metabolism pathway (Supplemental Figure 9).

**Figure 6:**
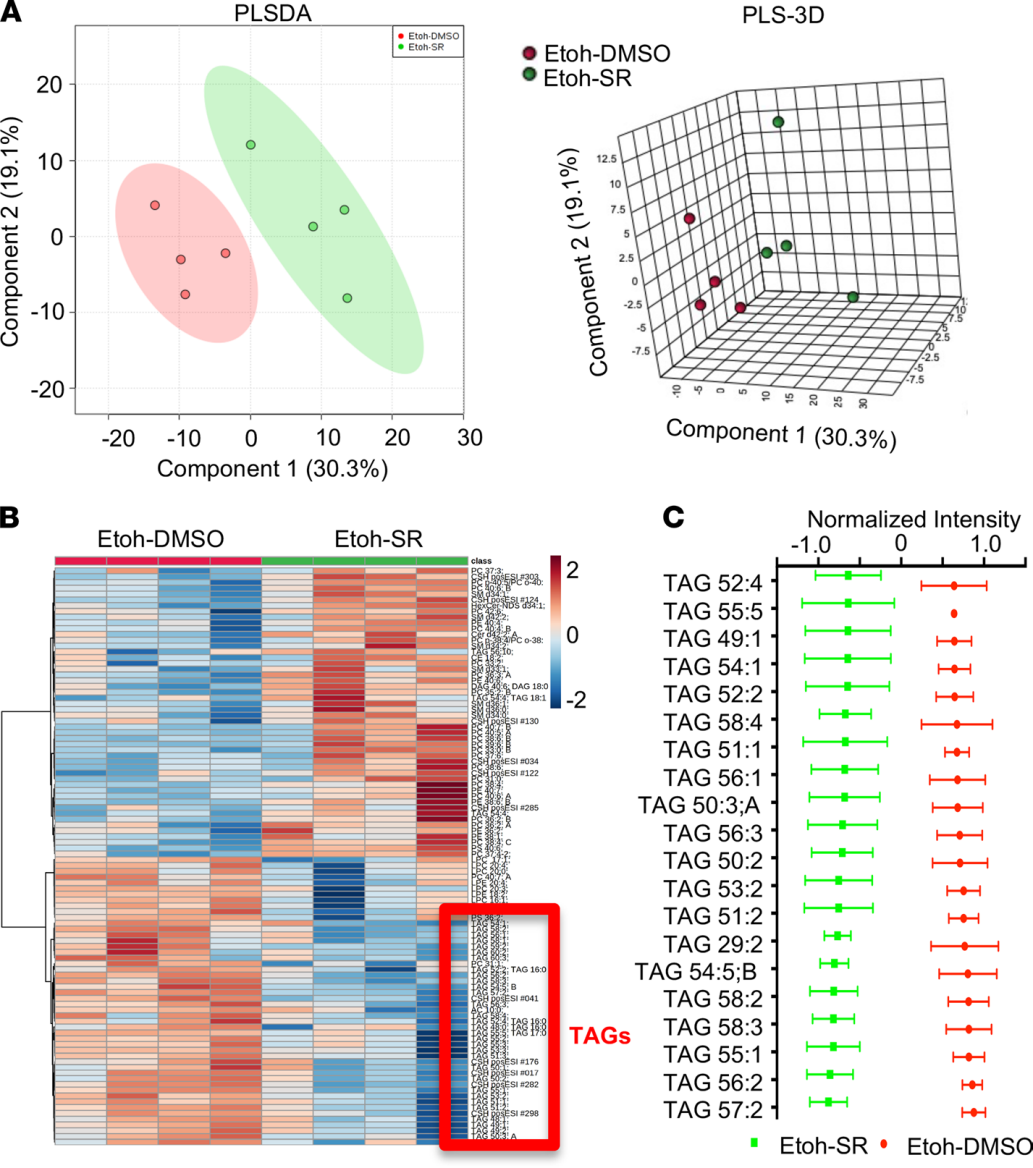
REV-ERBα agonist SR9009 treatment reduced hepatic TAGs in lipidomic profiles in ethanol-fed mice. (**A**) The predominance of partial least squares-discriminant analysis (PLS-DA) scores plots in 2D (left) and 3D (right) format. The ovals filled with different color indicated 95% CI Hotelling’s ellipses. (**B**) Heatmap showing differential abundance of lipids in ethanol-fed mice treated with SR9009 (Etoh-SR) versus ethanol-fed control mice (Etoh-DMSO). Red square indicated TAGs decreased in SR9009-treated ethanol-fed mice. (**C**) Top 20 significantly reduced TAGs in SR9009-treated ethanol-fed mice.

To focus on lipid metabolites, we subjected liver samples for lipidomic analysis. We observed significant alterations in hepatic lipidomes in ethanol-fed mice with and without SR9009 (Figure 6A). We found a significant reduction of hepatic triglycerides (TAGs) in mice treated with SR9009 (Figure 6B); the findings are consistent with the amelioration of hepatic steatosis (Figure 4). The top 20 significantly reduced TAGs were provided in Figure 6C. Our results indicated that the alterations in hepatic metabolic and lipidomic profiles upon alcohol treatment were partially restored with SR9009 treatment.

### CYP4A antagonist HET0016 rescued ethanol and REV-ERBα deletion induced lipid accumulation.

**Figure 7:**
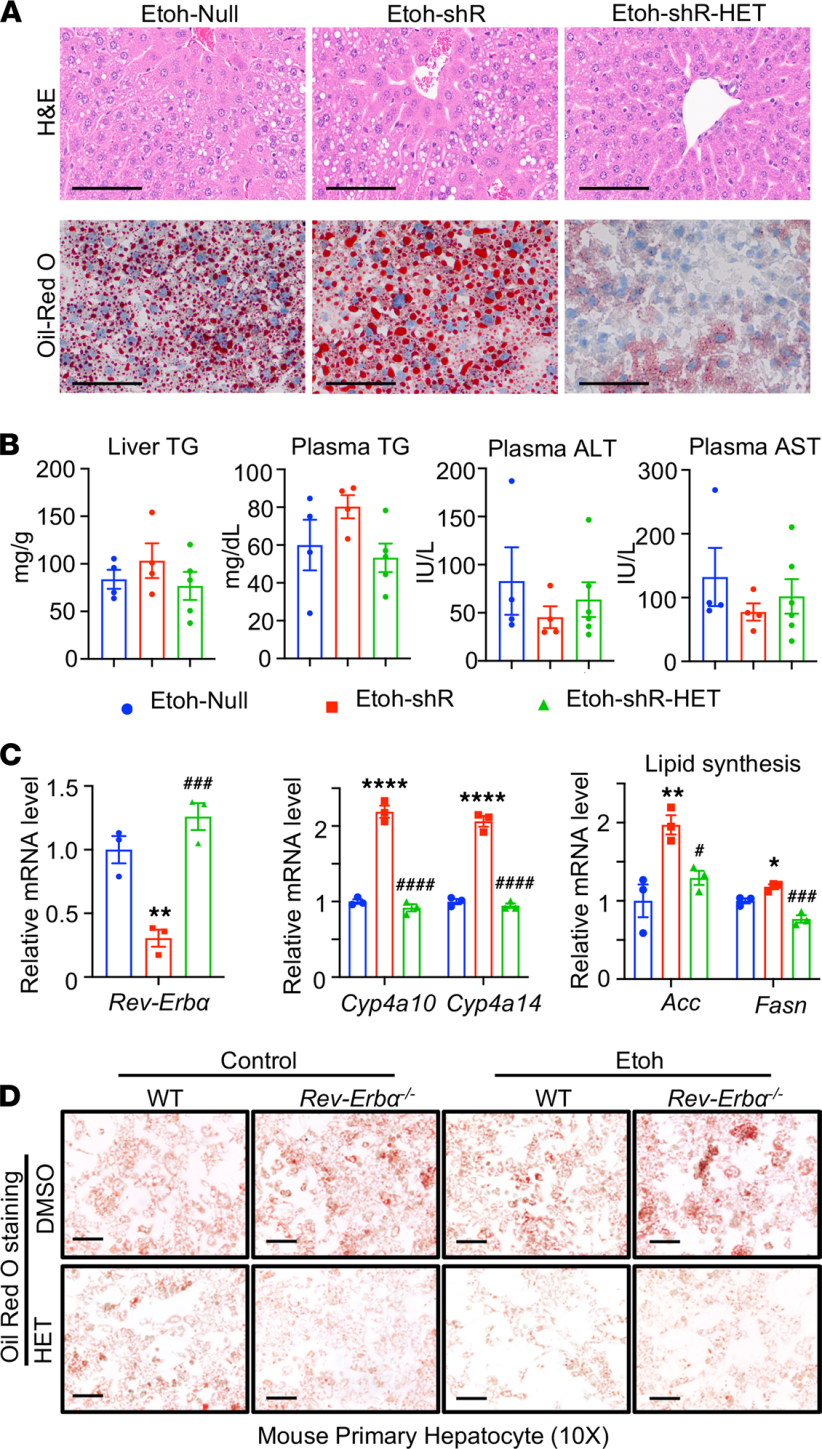
CYP4A antagonist HET0016 attenuated Rev-Erbα deficiency enhanced lipid accumulation in ethanol treatment. (**A**) HE (top) and Oil Red O staining (bottom) in Etoh binged mice livers of indicated groups. WT mice were injected with Null (Etoh-Null) or shRNA (Etoh-shR) for Rev-Erbα for 1 week before being subjected to the Etoh-binge model. During the Etoh feeding period, mice were IP injected with DMSO or HET0016 (HET, 5 mg/kg) (Etoh-shR-HET) daily. Scale bar: 200 μm. (**B**) Liver TG and plasma TG, ALT, and AST levels in indicated groups. (**C**) qPCR analysis of *Rev-Erb*α*, Cyp4a10,* and *Cyp4a14,* and *Acc* and *Fasn* mRNAs. *P < 0.05; **P < 0.01; ****P < 0.0001 versus Etoh-Null; ^#^*P <* 0.05; ^###^*P <* 0.001; ^####^*P <* 0.0001 versus Etoh-shR. One-way ANOVA. (**D**) Oil Red O staining in primary hepatocytes of WT or *Rev-Erb*α*−/−* mice. Primary hepatocytes were pretreated with HET0016 (4 μM) for 6 hours followed by 50 mM Etoh for 24 hours. Scale bar: 100 μm.

We next performed experiments to determine if blocking CYP4A is adequate in preventing alcoholic steatosis in REV-ERBα deficient state. Hepatic REV-ERBα knockdown was achieved by injecting mice with adenovirus-mediated delivery of sh-Rev-Erbα (shR). We observed an increase in plasma TGs, hepatic lipid accumulation, and genes involved in fatty acid synthesis in shR mice fed with ethanol; the observation was ameliorated with HET0016 treatment (shR-HET; Figure 7, A–C). As expected, we found an increase in the hepatic mRNA expression of *Cyp4a10* and *Cyp4a14* in ethanol-fed shR mice, and their expression was reduced in the presence of HET0016 treatment (Figure 7C).

We also performed in vitro experiments by pretreating primary hepatocytes from WT and *Rev-Erbα−/−* mice with either DMSO or HET0016 (4 μM) for 6 hours followed by ethanol at 50 mM for 24 hours. We found that HET0016 treatment significantly reduced lipid accumulation in *Rev-Erbα−/−* hepatocytes treated with ethanol compared with those treated with DMSO (Figure 7D and Supplemental Figure 10). Its effect was also confirmed using human hepatocyte cell line HC04 (Supplemental Figure 11). Our data confirmed that targeting CYP4A is an attractive therapeutic strategy for alcohol-induced liver injury.

### Increased expression of CYP4A in patients with alcoholic liver disease.

**Figure 8:**
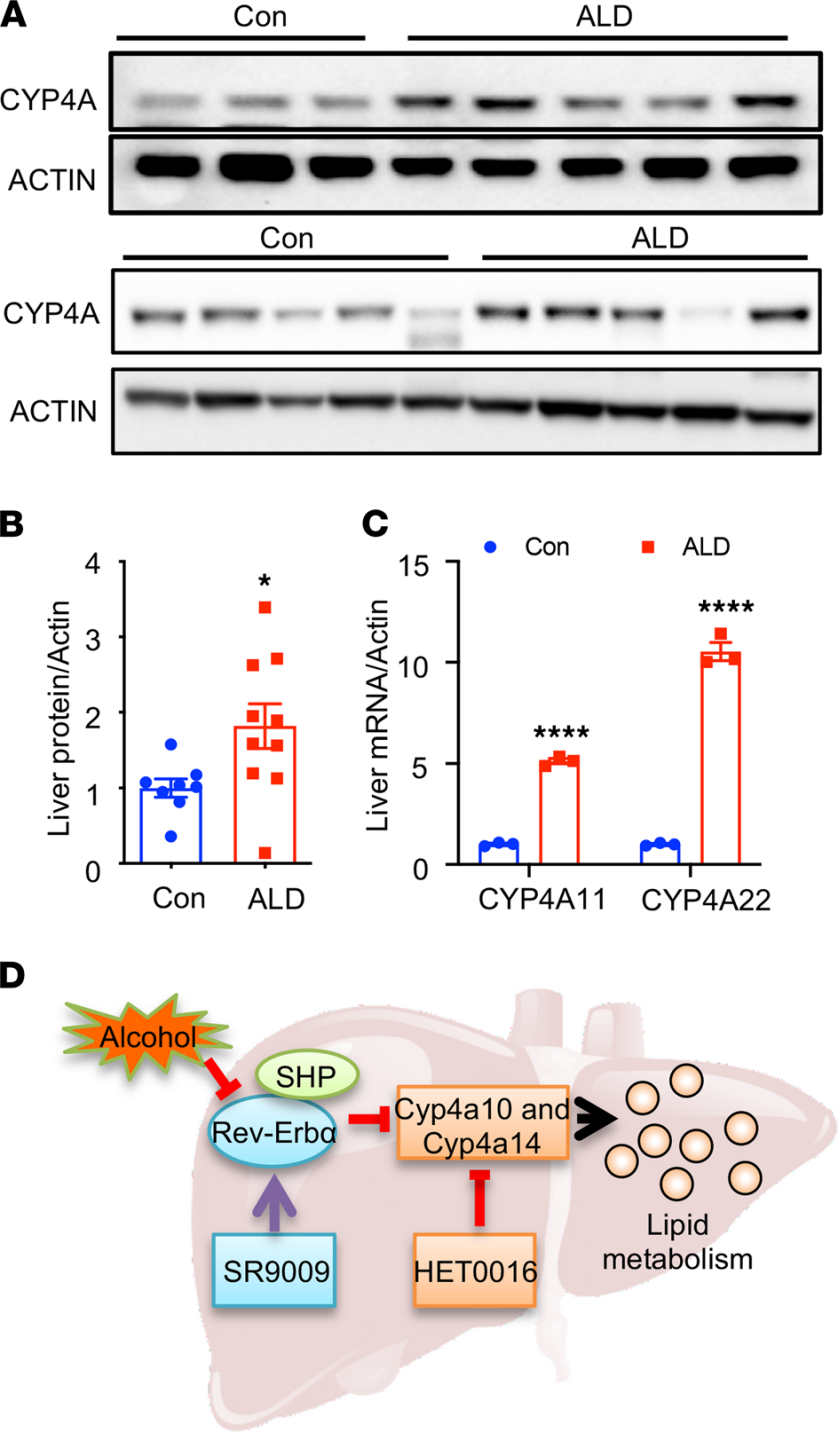
CYP4A was induced in human alcoholic liver cirrhosis. (**A**) Western blot analysis of CYP4A protein levels in controls and patients with alcoholic liver disease (ALD). ACTIN was used as the loading control. (**B**) The intensity scan of the Western blot bands using ImageJ (Version 2.0.0). *P < 0.05 versus controls. Student’s *t* test. (**C**) qPCR analysis for *CYP4A11* and *CYP4A22*, the human homolog of the mouse *Cyp4a14* and *Cyp4a10*, in human patients with ALD. ALD (*n =* 10, triplicated after pooled); Con, controls (*n =* 8, triplicated after pooled). ****P < 0.0001 versus Control. Student’s *t* test. (**D**) Proposed mechanism of the SHP/REV-ERBα/CYP4A axis in the pathogenesis of ALD. Alcohol activates CYP4A through the inhibition of REV-ERBα leading to hepatic steatosis and liver injury. Treatment with REV-ERBα agonist SR9009 or CYP4A antagonist HET0016 ameliorated alcohol-induced liver injury.

To determine if CYP4A is involved in ALD pathogenesis in humans, we detected the mRNA expression of *CYP4A11* (homolog of murine Cyp4a14) and *CYP4A22* (homolog of murine Cyp4a10) in the liver of patients with alcoholic liver disease. The baseline demographic and clinical characteristics of those patients were provided in Supplemental Table 1. We found a significant induction of *CYP4A11* and *CYP4A22* mRNA and protein levels in patients with ALD (Figure 8, A and B).

We also explored CYP4A11 and CYP4A22 expression in the liver of patients with cirrhosis using a recently published and publicly available data set (34). We found that both CYP4A11 and CYP4A22 were highly expressed specifically in hepatocytes compared with nonhepatic parenchymal cells, and that levels increased in patients with cirrhosis compared with healthy controls (as shown in Supplemental Figure 12). Our results suggest the importance of CYP4A activation in the pathogenesis of ALD.

## Discussion

We previously reported that the circadian timekeeping system is disturbed in alcoholic steatosis and that the effects of alcohol upon clock machinery contribute to the development of alcohol-induced liver injury (11, 35). However, the underlying mechanism has not been elucidated. CYP4A is a hydroxylase enzyme, which plays an important role in ω-hydroxylation primarily of medium- and long-chain fatty acid (21, 23). A previous study has shown the important function of CYP4A in the pathogenesis of nonalcoholic steatohepatitis (24); however, its function in alcohol-liver injury remains largely unknown. In our study, we found the induction of CYP4A in mice fed with ethanol and in patients with alcoholic liver disease. Interestingly, *Cyp4a10* and *Cyp4a14* mRNA expression was significantly reduced in ethanol-fed *Shp^−/−^* mice. Using available RNA-Seq data, we found that REV-ERBα, a transcription factor that is regulated by SHP, acts as the transcriptional repressor of *Cyp4a10* and *Cyp4a14*. This potentially novel finding was validated using ChIP assay. We further found that therapeutic intervention of REV-ERBα and CYP4A ameliorates alcohol-induced liver injury. Taken together, our study provides insight into the mechanism of the SHP/REV-ERBα/CYP4A axis in the pathogenesis of alcoholic liver disease.

REV-ERBα regulates the expression of its target genes in a circadian manner (27). REV-ERBα functions as a transcriptional repressor owing to the absence of coactivator-binding domain, activation-function (36). Several studies have shown the important role of REV-ERBα in the regulation of lipid metabolism (12, 37). *Rev-Erb*α^−*/*−^ mice had an elevation of hepatic apolipoprotein C3 gene expression and serum triglycerides (12). REV-ERBα also involves in the regulation of sterol regulatory element-binding protein (SREBP, encoded by gene *Srebf1*), a key transcription regulator in fatty acid synthesis (38). In our study, we also found the increase in lipid accumulation in *Rev-Erb*α*−/−* primary hepatocytes when compared with WT hepatocytes (Figure 3) and upon treatment with SR9009, a REV-ERBα agonist*,* the expression of *Srebf1* and fatty acid synthase (*Fasn*) was decreased in ethanol-fed mice (Figure 4D). In addition to lipid metabolism, REV-ERBα has been shown to regulate glucose metabolism through the regulation of glucose 6-phosphatase and phosphoenolpyruvate carboxykinase (39). We found the dysregulation of several metabolites in glucose metabolism and lipidomic pathway in the liver of ethanol-fed mice (Figure 5 and Supplemental Figure 9). Interestingly, the levels of these metabolites trended toward WT controls upon treatment with REV-ERBα agonist SR9009, providing evidence of REV-ERBα regulating glucose metabolism.

We found REV-ERBα as a transcriptional repressor for *Cyp4a10* and *Cyp4a14*, based on bioinformatic data and confirmatory experiments using ChIP analysis. Our data also confirmed a recent report demonstrating CYP4A as the target of REV-ERBα (40). *Cyp4a14* has been shown to play an important role in the development of nonalcoholic steatohepatitis (24). *Cyp4a14* deficiency markedly attenuated not only lipid accumulation but also hepatic inflammation and fibrosis in a mouse model of methionine and choline-deficient diet-induced NASH (24). Although the mechanism of CYP4A induction in alcohol-induced liver injury remains unclear, a previous study suggested CYP4A as a microsomal catalyst for oxidative stress (41). We also found evidence that CYP4A increased reactive oxygen regeneration (Figure 3E) and that inhibition of CYP4A using its antagonist, HET0016, significantly reduced CYP2E1 expression in mice fed with ethanol (Figure 4).

Our study also provided potentially novel information on the role of REV-ERBα and CYP4A as potential therapeutic targets for the treatment of alcohol-induced liver injury. We found that REV-ERBα agonist (SR9009) and CYP4A antagonist (HET0016) ameliorated liver injury in our mouse model (Figure 4). Mice that were treated with either SR9009 or HET0016 had lower levels of serum ALT and decreased expression of fatty acid synthesis gene. Our therapeutic studies also confirmed that CYP4A was the downstream target of REV-ERBα, based on the evidence that inhibition of CYP4A is adequate in preventing alcoholic steatosis in REV-ERBα deficient state (Figure 7).

In summary, we reported a mechanism linking the circadian pathway and alcohol-induced liver injury by identifying a possibly novel SHP-REV-ERBα-CYP4A axis in the pathogenesis of alcoholic liver disease. Activation of REV-ERBα and inhibition of CYP4A markedly attenuated hepatic steatosis and liver injury from alcohol. REV-ERBα and CYP4A are potential therapeutic targets for alcoholic liver disease.

## Methods

### Animals.

*Shp*^−/−^ mice were generated as previously described (42). *Rev-Erb*α*+/−* mice were purchased from the Jackson Lab and bred to generate *Rev-Erb*α*−/−* mice. Eight-week-old male mice (weight more than 20 g) were subjected to the National Institute on Alcohol Abuse and Alcoholism-ethanol plus binge model (26). Mice were sacrificed at 9 hours after gavage, when blood samples and liver tissues were collected (ZT 12), and then at every 6 hours (ZT 18, ZT 0, and ZT 6) over a 24-hour period.

In selected experiments, in vivo overexpression of flag-tagged REV-ERBα was performed using TurboFect in vivo Transfection Reagent (Thermo) as described previously (18). For in vivo knockdown of REV-ERBα, mice were injected through the tail vein with 1 × 10^11^ AAV8-TBG-Null or AAV8-TBG-U6-shREV-ERBα virus for 2 weeks. Then, they were fed using ethanol plus binge protocol. At the end of the experiments, liver tissues were collected at 9 hours after an oral ethanol binge.

The schematic diagram for the administration of REV-ERBα agonist (SR9009) or CYP4A antagonist (HET0016) is shown in Supplemental Figure 7. The REV-ERBα agonist (SR9009 at 100 mg/kg/d; ref. 29) and CYP4A antagonist (HET0016 at 5 mg/kg/d; ref. 31) were dissolved in DMSO and injected i.p. daily during the ethanol feeding period. At the end of the experiments, serum and liver tissue were harvested at 9 hours after oral ethanol binge.

### Cell line and in vitro transfection.

The human hepatocyte cell line HC-04 (provided by José Manautou, University of Connecticut) was maintained in equal volumes of DMEM and Ham’s F-12 media (Gibco) supplemented with 10% FBS (Gibco). HEK293T (CRL-3216), AML12 (CRL-2254) or Hepa 1 (CRL-1830) cells (ATCC) were maintained in DMEM with 10% FBS, 100 IU/mL penicillin G, and 100 μg/mL streptomycin (Invitrogen). They were incubated in a 37°C in a humidified incubator with a 5% CO_2_ atmosphere. Transfection experiments were performed using X-tremeGENE HP DNA transfection reagent (Roche). The luciferase reporter assay was performed using Dual-Glo Luciferase Assay System, as described previously (18). The luciferase activity was normalized to Renilla luciferase activities in the same sample.

### PLA and IP.

The cells were fixed with 4% paraformaldehyde in 1× PBS for 15 minutes. followed by blocking with 5% normal goat serum with 0.3% Triton X-100 in PBS. Mouse anti-FLAG (F1804, MilliporeSigma) and rabbit anti-GFP (SAB4701015, MilliporeSigma) were used to incubate the slices overnight for detecting the interaction between SHP and REV-ERBα. In situ PLA was carried out using Duolink In Situ Red Starter Kit mouse/rabbit (MilliporeSigma) according to the manufacturer’s protocol. The images were taken by Nikon A1R confocal laser microscope (Nikon). For the IP, HEK 293 cells were transfected with indicated plasmids for 24 hours before the treatment. Then, cells were lysed with Pierce IP lysis buffer (Thermo) and the complex was pulled down with the anti-flag M2 magnetic beads (MilliporeSigma). The interacted protein was detected with mouse anti-FLAG (MilliporeSigma) and rabbit anti-GFP Abs (MilliporeSigma).

### Primary hepatocytes isolation and treatment.

Primary hepatocytes isolation and culture were described previously (43). Briefly, the mice were anesthetized, and the liver was perfused with a prewarmed solution. The hepatocytes were suspended in 50% Percoll (MilliporeSigma), and seeded on collagen-coated culture plates with William E medium (MilliporeSigma) containing 2 mM glutamine, 1% Pen/Strep, and 5% FBS. Four to six hours after cell attachment, the medium was changed with William E medium without FBS before treatment with ethanol and other treatments, as indicated.

### ChIP assay.

Liver tissues were minced and cross-linked by 1% formaldehyde for 20 minutes before quenching with 1:20 volume of 2.5 M glycine solution for 5 minutes. After washing with PBS twice, the nuclear were extracted by Dounce homogenization in ChIP buffer (50 mM Tris-HCl, pH 7.5, 1 mM EDTA, 140 mM NaCl, 0.1% sodium deoxycholate, 1% Triton X-100). The chromatin fragments were prepared in lysis buffer (50 mM Tris-HCL, pH 8.0, 10 mM EDTA, 0.1% SDS) by sonication. Proteins were immunoprecipitated using REV-ERBα Ab (13418, CTS, MA) or IgG in ChIP buffer. The cross-link was reversed overnight in SDS buffer (50 mM Tris-HCL, pH 8.0, 10 mM EDTA, 1% SDS), and DNA was purified and used as templates for qPCR.

### Luciferase assay.

AML12 or Hepa1 cells (ATCC CRL-2254, ATCC CRL-1830) were transfected with mouse *Cyp4a10* or *Cyp4a14* promoters (sequences were provided in Supplemental Figure 5), which was cloned into pGL3-Basic (Promega) and sequenced for confirmation. The transfections were performed using Lipofectamine 2000 (Thermo). Luciferase activities were detected using Dual-Luciferase Reporter Assay (Promega) and normalized against renilla activities. Triplicates were performed in each group, and each experiment was repeated 3 times.

### IHC, Oil Red O, and Nile Red staining.

Liver tissues were collected and fixed in 10% formalin on a shaking device for 24–48 hours, paraffin-embedded, and sliced into 5 μm sections for the following H&E staining according to the standard protocol. For IHC, Abs against CYP4A (sc-271983) and CYP2E1 (AB1252, Millipore) were used to stain the paraffin sections and visualized with DAB Peroxidase Substrate Kit (Vector Laboratories). For the Oil Red O staining, 5 μm frozen sections were prepared by cryosection from snap-frozen liver tissues. The primary hepatocytes or human hepatocyte cell line HC04 was treated and fixed with 4% paraformaldehyde. The sections or cells were stained in 0.5% Oil Red O in 60% isopropanol for 30 minutes. The images were taken by Olympus BX41 microscope. Cultured cells or frozen sections were fixed with 4% paraformaldehyde and stained with 250 μg/mL Nile Red solution for 15 minutes. The images were obtained by Nikon A1R confocal laser microscope (Nikon).

### Liver TG assay and ALT/AST assay.

Serum and liver TG were analyzed using Pointe Scientific Triglycerides Liquid Reagents (Thermo) as described previously (18). Serum ALT and AST were measured using Infinity ALT and AST reagents (Thermo) according to the manufacturer’s instructions. All experiments were performed in duplicates.

### Measurement of ROS generation.

ROS generation in primary hepatocytes was measured using 2′,7′–dichlorofluorescin diacetate (DCFDA) Cellular Reactive Oxygen Species Detection Assay Kit (Abcam) as previously (44). Briefly, primary hepatocyte from WT or *Rev-Erb*α*−/−* mice livers were isolated and seeded to the black wall, clear-bottom 96-well microplate overnight before the indicated treatments; DCFDA solution was added and incubated indicated time. The fluorescence was measured with excitation at 485 nm and emission at 535 nm using a microplate reader (Bio-Tek).

### Quantitative PCR.

Total RNA was isolated using TRIzol Reagent (Invitrogen) and cDNA synthesis was performed with High-Capacity cDNA Reverse Transcription Kit (Applied Biosystems). Quantitative PCR (qPCR) was performed with iTaq Universal SYBR Green Supermix (Bio-Rad). The primers were provided in Supplemental Table 2. Each qPCR analysis was run in triplicate. The relative ratio of the indicated genes was normalized to internal control, β-actin.

### Western blots.

Human or mouse liver tissues or primary mouse hepatocytes were lysed using RIPA buffer (0.5 M Tris-HCl, pH 7.4, 1.5 M NaCl, 2.5% deoxycholic acid, 10% NP-40, and 10 mM EDTA) with protease inhibitors (MilliporeSigma). The Pierce BCA Protein Assay Kit (Thermo) was used to measure the concentration of protein. The standard procedures of SDS-PAGE were performed using 30 μg lysates and transferred to nitrocellulose membranes. The SuperSignal West Pico Chemiluminescent Substrate (Thermo) was used according to the manufacturer’s protocol. The following Abs were used: CYP4A (sc-271983; Santa Cruz Biotechnology), ACTIN (sc-47778; Santa Cruz Biotechnology), and CYP2E1 (AB1252; Millipore).

### Metabolomic analysis.

Metabolomic and lipidomics analyses were performed at the West Coast Metabolomics Center, University of California, Davis, as described previously (43). Briefly, 50 mg frozen liver tissues were submitted for gas chromatography/mass spectrometry (GC/MS) to detect the primary metabolism. Leco ChromaTOF software (Version 2.32) was used for data preprocessing. Another 50 mg frozen liver tissues were submitted for complex lipids by CSH-QTOF MS/MS analysis. The online web-based software, MetaboAnalyst (https://www.metaboanalyst.ca/), was used for statistical, functional, and integrative analysis (45).

### RNA-Seq and bioinformatics analysis.

Sequencing was performed using the Illumina TrueSeq RNA Library Preparation Kit v2 with polyA selection to obtain 50 cycles single-end reads. The reads were aligned to the December 2011 mouse reference sequence genome (GRCm38) using the Novoalign short-read alignment software (Version 1.0A). Sample reads were visualized using the Integrated Genome Browser (Version 9.0.1). Differentially expressed genes were identified using the Useq software package from the University of Utah. The DRDS function calculated the false discovery rate (FDR) statistic for the significance of differentially expressed genes. Log-transformed FPKM of >0.1 in at least one treatment group were used for the analysis. We only used differentially expressed genes (DEGs) with fold changes greater than 1.5 and with a log-transformed FDR of 0.05 or less. Means-centered log-transformed FPKM were used to make hierarchical clustering heatmaps in Cluster (version 3.0) and Java Tree View (version 3.0). The differentially expressed genes were analyzed and networks and pathway comparison diagrams were generated using QIAGEN’s Ingenuity Pathway Analysis (IPA, Hilden; www.qiagen.com/ingenuity). The original sequencing data were submitted to National Center for Biotechnology Information with an access number, GSE137059. The upregulated genes in *Rev-Erb*α*−/−* mice liver were gated with a fold change of more than 1.3 in both GRO-Seq (GSE59486) and microarray (GSE59460, FDR<0.1) experiments (27). The downregulated genes in *Shp^−/−^* mice liver were obtained from GSE43893 (FDR<0.05; fold change>1.5; ref. 46). The Rev-Erbα ChIP-Seq analysis was obtained from GSE67962 (27). The single-cell sequencing data from human cirrhotic livers were obtained from GSE136103. The plots were downloaded from an open-access gene browser (http://www.livercellatlas.mvm.ed.ac.uk) (34).

### Statistics.

All experiments were performed in triplicate. The data are shown as the mean ± SEM. Comparisons between groups were performed using 2-tailed Student’s *t* test or 2-way ANOVA with post hoc analysis (Prism Version 7.0) for the continuous variables. A *P* value of less than** 0.05 was considered significant.

### Study approval.

The deidentified human liver specimens for mRNA and protein analysis were collected under an IRB-approved protocol at the Indiana University–Purdue University Indianapolis and the Liver Tissue Procurement and Distribution System (Minneapolis, Minnesota, USA) as described previously (47). All animal experiments were performed in accordance with relevant guidelines, and regulations were approved by the IACUC at Indiana University–Purdue University Indianapolis and the University of Connecticut.

## Author contributions

ZY and SL originated the study concept and design. ZY, RVS, YH, YJ, PK, TZ, SH, WB, DAD, NJS, JM, and NH managed data acquisition. ZY, RVS, LW, DAD, and SL analyzed and interpreted data. NJS critically revised the manuscript. ZY and SL drafted and finalized the manuscript. All authors have read and approved the manuscript for submission. The order of the co–first authors in the author list was determined as follows: ZY participated in the study since the study inception and planning stage, and RVS assisted with experiments to complete the study.

## Supplementary Material

Supplemental data

## Version 1 08/23/2021

Electronic publication
