# Supplementary material for: The role of SHP/REV-ERBα/CYP4A axis in the pathogenesis of alcohol-associated liver disease
Source: JCI Insight. 2021 Aug 23;6(16):e140687. doi: 10.1172/jci.insight.140687 (PMC8410014; doi:10.1172/jci.insight.140687)

Etoh Rev-Cyp, Supp. Fig. S1

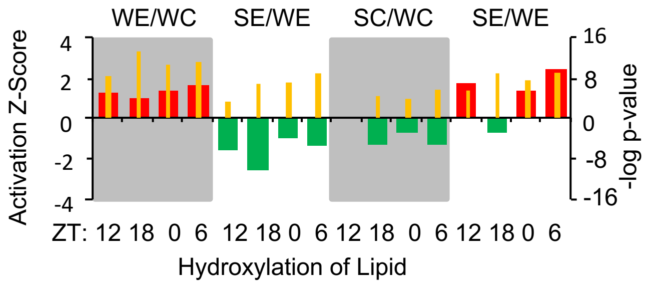

Supp. Fig. S1 IPA activation scores at all ZT using Ingenuity Pathway Analysis (IPA). Red: up-regulated in indicated comparisons; Green: down-regulated in indicated comparisons; Yellow: p-value for enrichment of the lipid hydroxylation pathway at each ZT (Fisher's exact test).

Etoh Rev-Cyp, Supp. Fig. S2

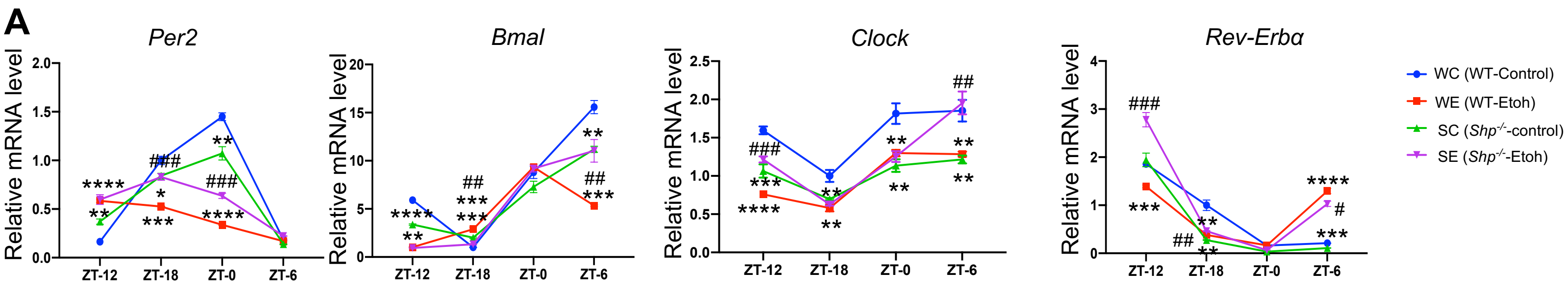

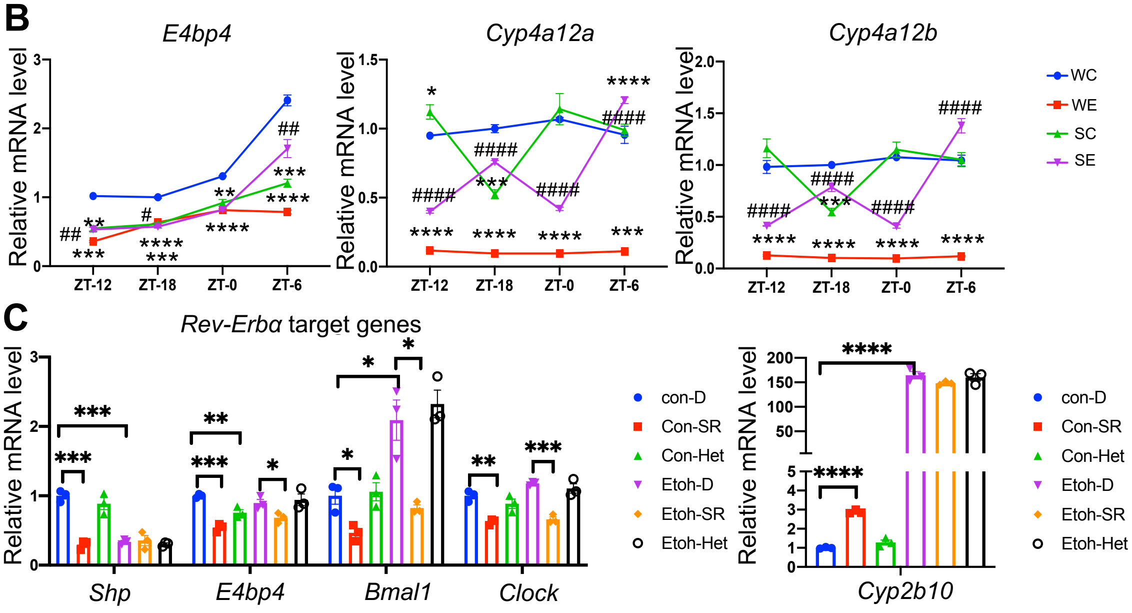

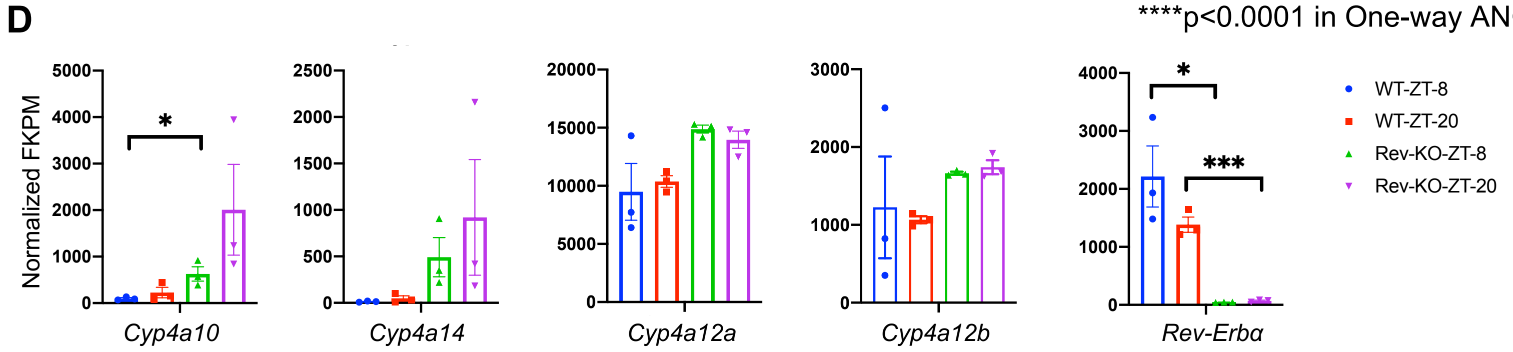

Supp. Fig. S2 Rhythmicity of core clock genes and *Rev-Erbα* target genes expression levels in the alcoholic liver. (A-B) qPCR analysis of core clock genes (*Per2, Bmal and Rev-Erbα*), *Rev-Erbα* target genes (*Bmal, E4bp4*) and) in WT and *Shp*$^{-/-}$ mice liver*Cyp4a12 (Cyp4a12a and Cyp4a12b)* fed with Etoh plus binge. *p<0.05, **p<0.01, ***p<0.001, ****p<0.0001 vs WC; $^{\#}$p<0.05, $^{\#\#}$p<0.01, $^{\#\#\#}$p<0.001, $^{\#\#\#\#}$p<0.0001 vs WE. Two-way ANOVA. **(C)** qPCR analysis of *Rev-Erbα* target genes (*Shp, E4bp4, Bmal, Clock and Cyp2b10*) in SR9009 (SR, REV-ERBα agonist) or HET0016 (HET, CYP4A antagonist) treated mice liver in Etoh-binge model. One-way ANOVA. **(D)** RNA-seq analysis of the rhythmic expression of *Cyp4a10 and Cyp4a14* in WT and *Rev-Erbα*$^{-/-}$ mice (Rev-KO) liver. The data was obtained from GSE79087. *: p<0.05, **: p<0.01,***:p<0.001, ****p<0.0001 in One-way ANOVA.

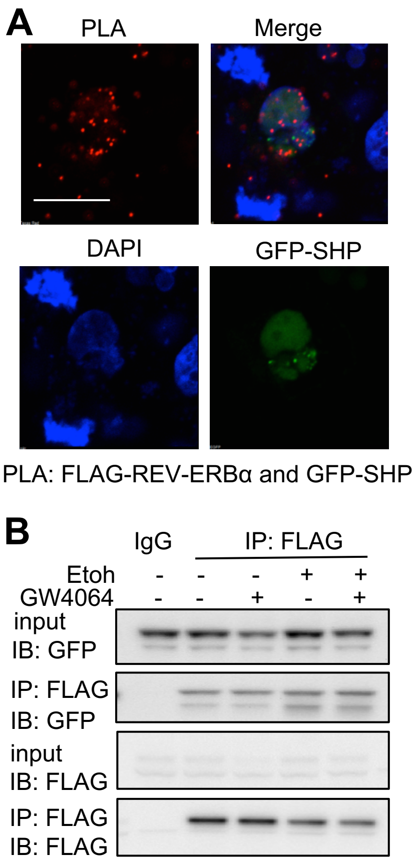

Supp. Fig. S3 Interaction between GFP-SHP and FLAG-REV-ERBα proteins located in the nucleus of HEK293T cells. (A) Proximity ligation assay (PLA) were performed using rabbit anti-GFP and mouse anti-FLAG antibodies. Red indicated the interacted SHP and REV-ERBα; Green indicated the expression of GFP-SHP. Scale bar: 50μm. **(B)** Immunoprecipitation (IP) between SHP and REV-ERBα in condition of ethanol and/or FXR agonist (GW4064) treatment. HEK 293T cells were transfected with FLAG-REV-ERBα and GFP-SHP for 24 hours and fasted for another 24 hours. Then the cells were pre-treated with 5nM GW4064 for 1 hours before treated 50mM Etoh overnight. The cell lysis were immunoprecipitated with anti-FLAG antibody. The anti-FLAG and anti-GFP antibodies were used for WB.

Etoh Rev-Cyp, Supp. Fig. S4

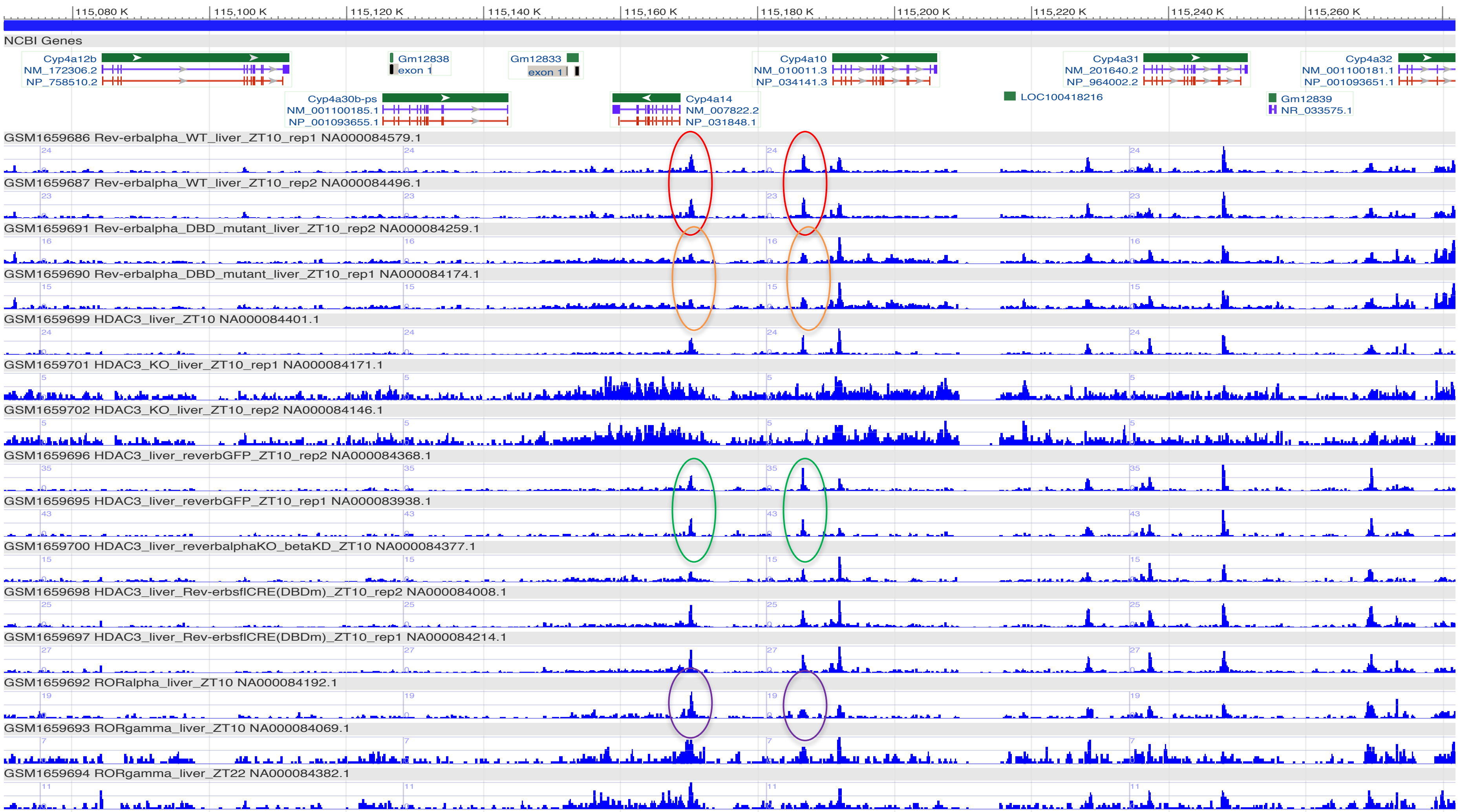

<u>Supp. Fig. S4</u> REV-ERBα binding on both Cyp4a10 and Cyp4a14 promoter from CHIP-seq analysis (GSE67962). The bindings (Red circles) were diminished when REV-ERBα DNA-binding domain was mutant (Orange circles). HDAC3 (Green circles) and RORalpha (Purple circles) bind to both promoter at same location.

Etoh Rev-Cyp, Supp. Fig. S5

>NC_000070.5:115186263-115186950 Mus musculus strain C57BL/6J chromosome 4, MGSCv37 C57BL/6J

Cyp4a10 pro

CAACCCCTGACAGGTTGACTATGTTCCAGTGAATGGCCACATAGGAATATATGTATTACACTAACTGGACTTGA
TAGGTTTAGGGTCACAAGAAGAAGACAAGGAATTCAGTCTAAAGGGAAATGGAGTAGAGATCTAGGAGGAAT
TGTGAGGGCATGAATATAGACAAAACATGTTGTATAACGTTTTTAAAAGATTCGTAACAGTAACTGACAAGCAG
ATTTAAATTCTAAAATTCTTTACAACATGTTCCTTTATTTTAAGGGTGACAAATGGGTTCTTGGATATAGAAGGA
AAAGGCCACCGTCTAAAAGTTCT**AGGTCA**GAGGAGAAGAGTACAATGTGGATAGAAAAAGTAGTCATGAAAC
TAGGGTAAAGTTCAGTGAGATGGAGTGTAAGTTATTCCATTGCCCTTTGCTCTAGTTTTTGACAGAGCATATG
ATTTGGGAGGAAAAAAAAAAGACCTTTGGCCCTAGGAGTTGAGAAATAAATCCCTTGTTATTAGAAAAGCAGA
GGAGAAGCCTTTGAGGGCATGTCAGCAGATTCTGGGGGAGGCAGCCTTCTAGTCCAATAGGGAAGGTTACC
AATACCACCATGGACTCTAAATGATTGTTTTCCTTTGAACAATATCACTTGGACTCTCTGTTATCAGTTTCTTAT
CACTGAAATAAGGGCAAGGGTAAAACCAA

mC4a10-pro-F: GGGGTACCCCAGTGAATGGCCACATAGGA (Kpn1)
mC4a10-pro-R: CCAAGCTTCTCCCCCAGAATCTGCTGAC (Hind3)
mC4a10-CHIP-F: GAAGGAAAAGGCCACCGTCT
mC4a10-CHIP-R: ACTCCTAGGGCCAAAGGTCT

>NC_000070.5:115170123-115170466 Mus musculus strain C57BL/6J chromosome 4, MGSCv37 C57BL/6J

CYP4a14 pro

CCCACTACTTTTTCTTTCTTTCCACAATTCTGAGCTTT*GGTCA*AAACCAAAGACCGAGAGGGACATGCTCCAT
ACGTCTGAACTTTTAGCCCACTTTGT*GGTCA*TTTTACAACTTCAAATATTTGTTGGATGAGTAAATGATTGAAA
GATCCAGTCCATTTCTTGGGAC**AGGTCA**TTTTGTACCCTTTCTCCTTCCCTACTTGGATCCTCTCCCACTTTC
AGAGTGGAATTCAGTAGTGGAAAGAGCTTTGGCGTCCAAGACTACTGCTGAATTTTAGTTTTGACTGTTTCCT
TCTCTGAATCTGAGTTCTTTTTGTTTGACCCATATTATGGTAAGCATGAAC

mC4a14-pro-F: GGGGTACCCCACAATTCTGAGCTTTGGTCA （Kpn1)
mC4a14-pro-R: CCAAGCTTTGGGTCAAACAAAAAGAACTCAGA (Hind3)
mC4a14-CHIP-F: GGGACATGCTCCATACGTCT
mC4a14-CHIP-R: ACGCCAAAGCTCTTTCCACT

Supp. Fig. S5 Sequences of cyp4a10 and cyp4a14 promoter around REV-ERBα binding peaks. Underlined sequences indicated the REV-ERBα binding sites. Primers for cloning those promoters and for CHIP assay were provided as labeled.

Etoh Rev-Cyp, Supp. Fig. S6

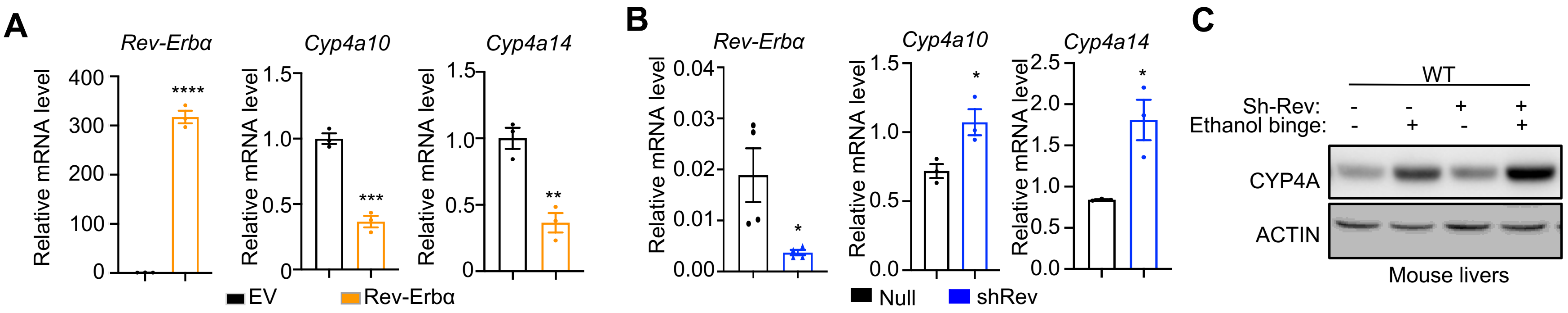

Supp. Fig. S6 REV-ERBα regulated CYP4A *in vivo*. (A-B) qPCR analysis of Rev-Erbα, *Cyp4a10* and *Cyp4a14* mRNAs in Rev-Erbα overexpressed mice liver (**A**) or Rev-Erbα knock down mice liver. T-test. (**B**). EV: Empty vector; shRev: shRNA for Rev-Erbα. *: p<0.05, **p<0.01, ***p<0.001, ****p<0.0001 vs EV or Null. T-test. (**C**) Western blot analysis of CYP4A protein level in indicated group.

Etoh Rev-Cyp, Supp. Fig. S7

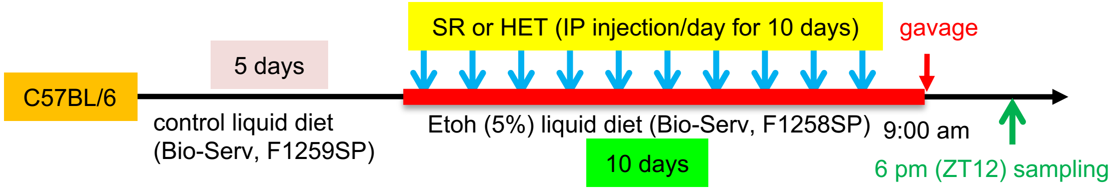

<u>Supp. Fig. S7</u> Schemes for agonist or antagonist application in Etoh-binge model. Rev-Erbα agonist (SR9009,100mg/kg/day) or Cyp4a antagonist (HET0016, 5mg/kg/day) was injected through IP daily in ethanol feeding period in Etoh-binge model.

Etoh Rev-Cyp, Supp. Fig. S8

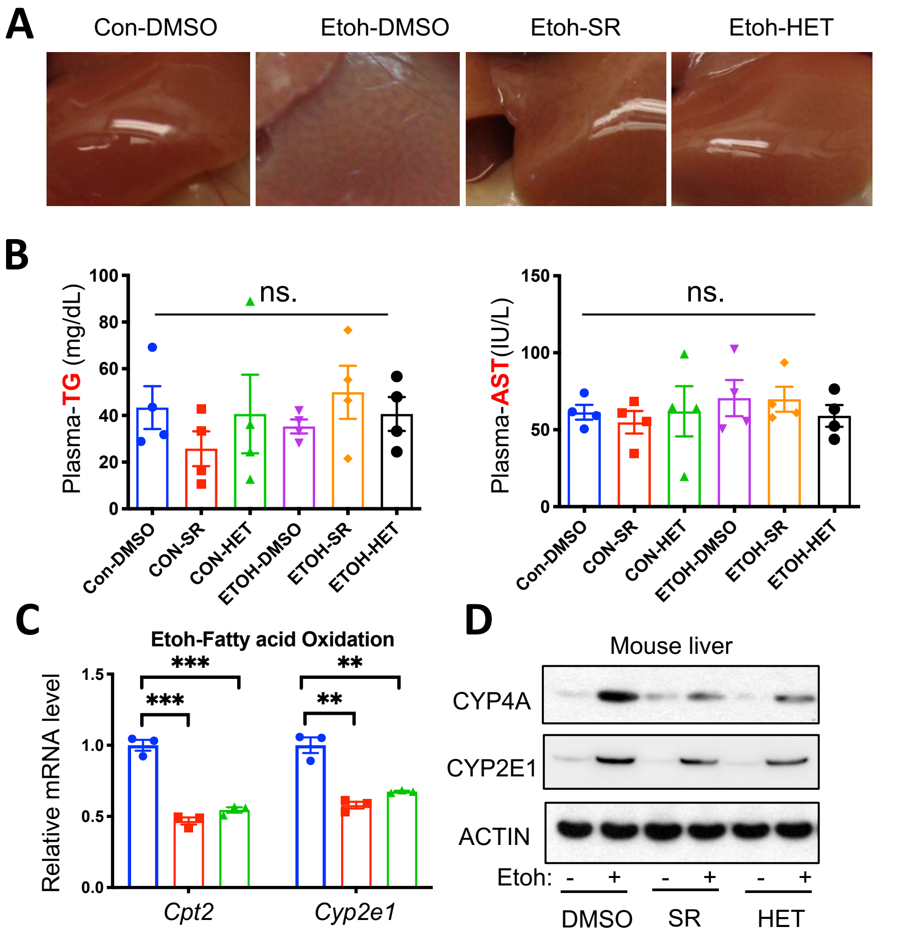

<u>Supp. Fig. S8</u> Treatment of REV-ERBα agonist SR9009 or CYP4A antagonist HET0016 significantly improved alcoholic steatosis and alcohol-induced liver injury. (**<u>A</u>**) Gross appearance of the liver surface. (n=4/group). (**<u>B</u>**) Serum TG and AST levels in indicated groups. One-way ANOVA. (**<u>C</u>**) qPCR analysis of genes involved in fatty acid oxidation. **p<0.01, ***p<0.001 vs Etoh-DMSO. One-way ANOVA. (**<u>D</u>**) Western blot analysis with CYP2E1 and CYP4A antibodies in each group. Con: control; Etoh: ethanol; SR: REV-ERBα agonist-SR9009; HET: CYP4A antigonist-HET0016.

Etoh Rev-Cyp, Supp. Fig. S9

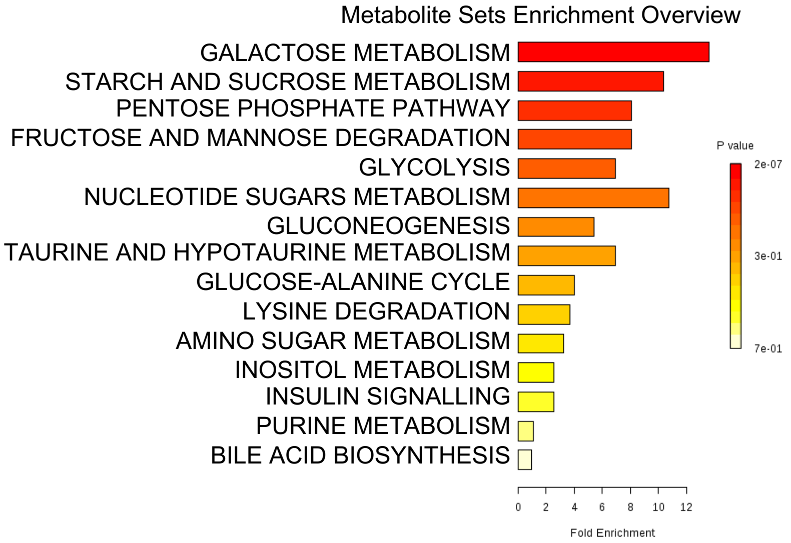

Supp. Fig. S9 The overview of Metabolite Sets Enrichment (MESA) in ethanol fed mice treated with SR9009 (Etoh-SR) vs ethanol fed mice (Etoh-DMSO).

Etoh Rev-Cyp, Supp. Fig. S10

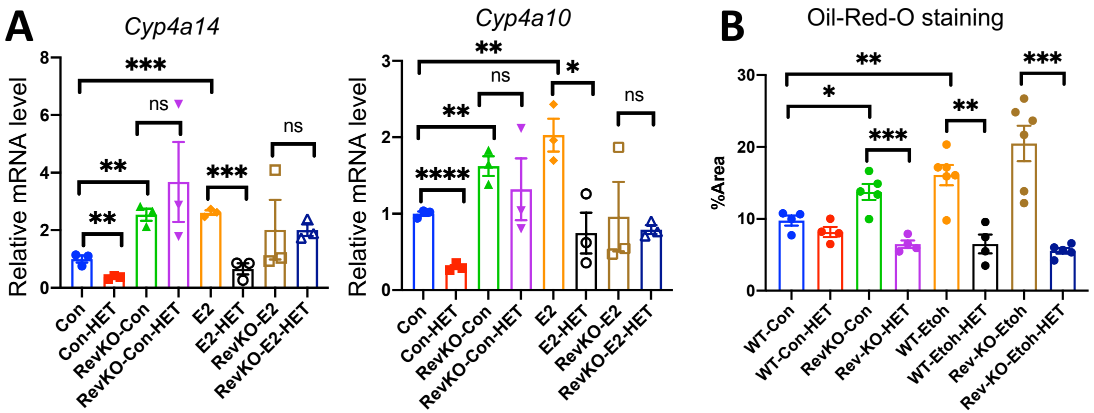

Supp. Fig. S10 Deficiency of Rev-Erbα blocked HET reduced Cyp4a14 and Cyp4a10. (A) qPCR analysis of Rev-Erbα, *Cyp4a10* and *Cyp4a14* mRNAs in WT or Rev-Erbα$^{-/-}$ (RevKO) mouse primary hepatocyte treated with E2 (50 mM) or HET (4nM) for 24 hours. One-way ANOVA. **(B)** Quantification of the positive area in Oil-Red-O staining for **Fig. 7D**. *: p<0.05, **p<0.01, ***p<0.001, ****p<0.0001 vs indicated group. One-way ANOVA.

Etoh Rev-Cyp, Supp. Fig. S11

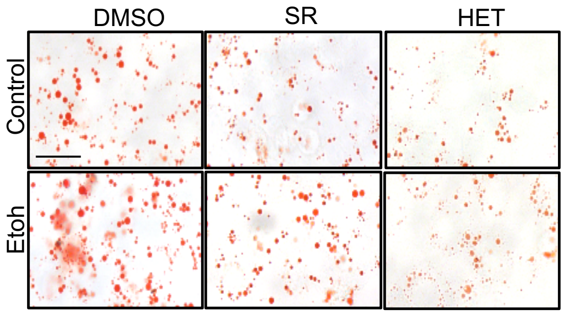

HC04 (human hepatocyte, (10X)

<u>Supp. Fig. S11</u> Oil-red O staining in human hepatocyte cell line HC04. HC04 cells were pre-treated with SR9009 (5μM) or HET0016 (4μM) for 6 hours followed by ethanol 50 mM (Etoh) for another 24 hours. Scale bar: 100μm.

Etoh Rev-Cyp, Supp. Fig. S12

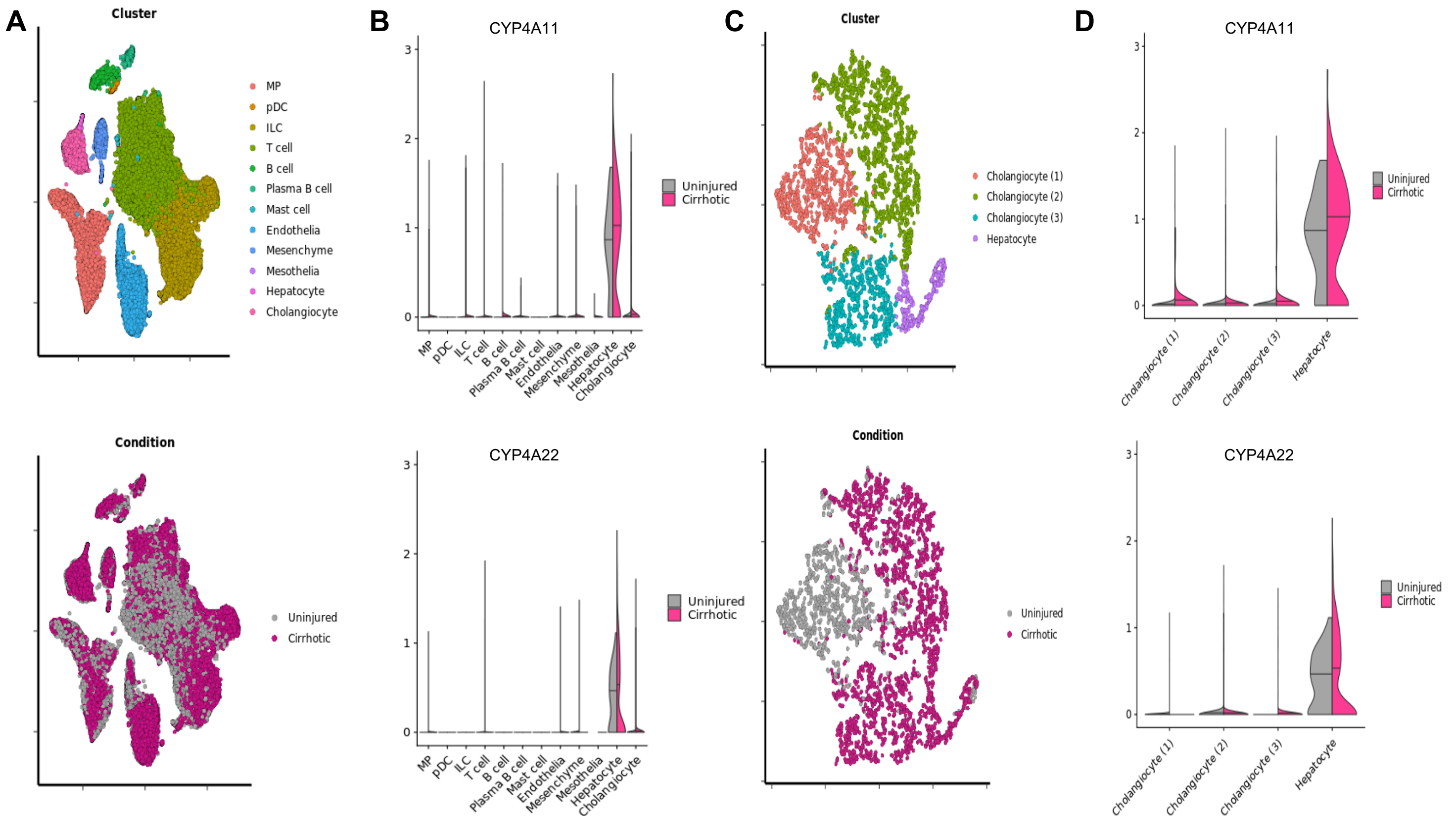

<u>Supp. Fig. S12</u> CYP4A11 and CYP4A22 expression in human cirrhotic livers at single cell level . (<u>A</u>) human liver cells from 5 healthy and 5 cirrhosis were isolated using FACS sorting and subjected for single cell sequencing. The cells were clustered and the color indicated the cell types or samples as shown in figure. **(<u>B</u>)** The expressions of CYP4A11 and CYP4A22 were high only in hepatocyte compared to other cell types. **(<u>C</u>)** The clustering of epithelia cells (upper) and distribution of samples (bottom). **(<u>D</u>)** The expression levels of both CYP4A11 and CYP4A22 were increased in cirrhotic liver hepatocytes. The plot were downloaded from and open-access gene browser (http://www.livercellatlas.mvm.ed.ac.uk) and the raw data is available in GSE136103.

Etoh Rev-Cyp, Supp. Fig. S13

Fig. 1

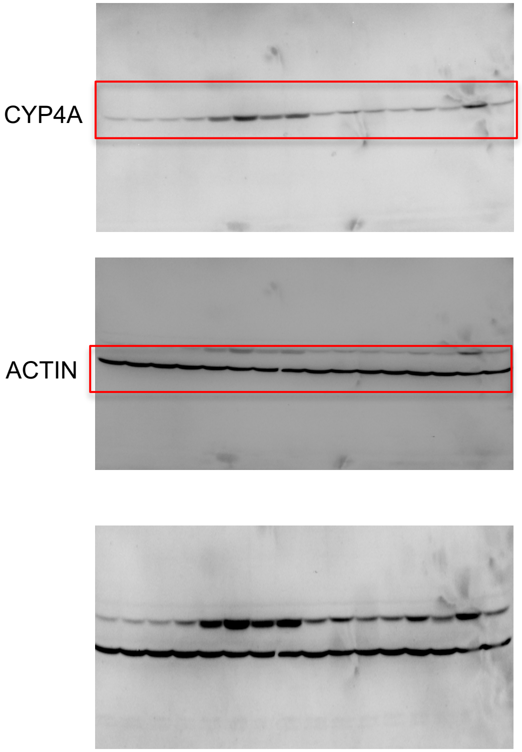

Fig. 3

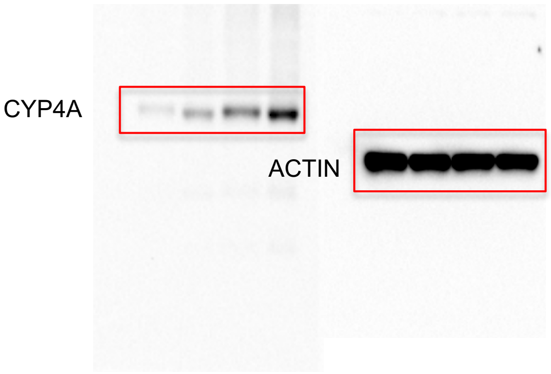

Fig. 8

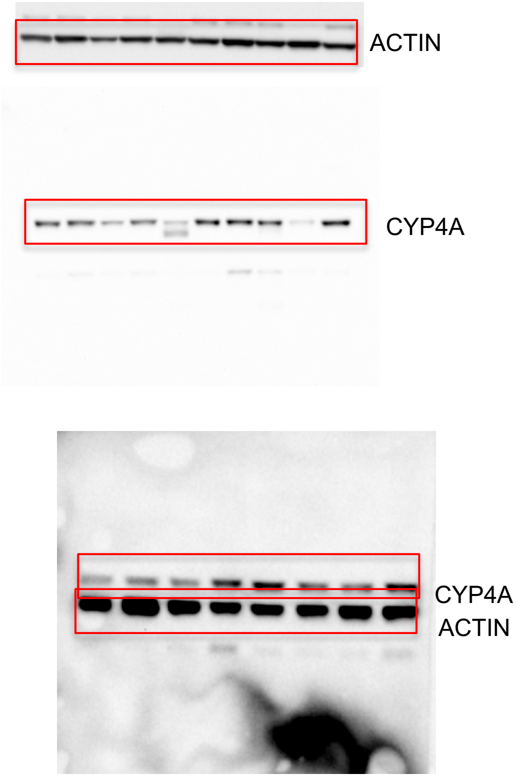

<u>Supp. Fig. S13</u> Original blot for the Western blot.

Etoh Rev-Cyp, Supp. Fig. S13 continue

Supp. Fig. S3

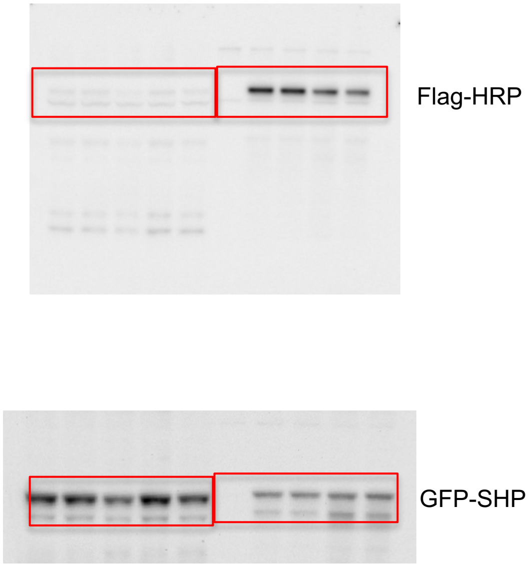

Supp. Fig. S6

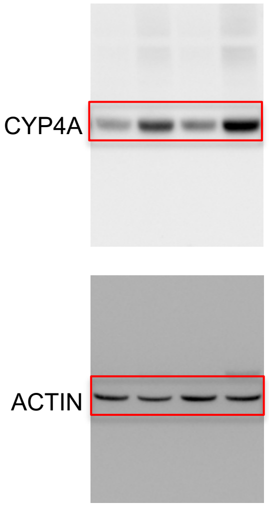

Supp. Fig. S8

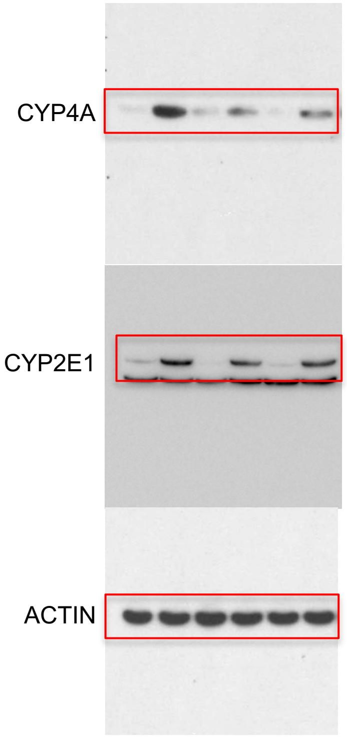

Supplement: Supplemental data [file jciinsight-6-140687-s153.pdf]
